# *FOSL2* regulates endothelial cell state and chromatin accessibility in systemic sclerosis pulmonary vascular remodeling

**DOI:** 10.1172/jci.insight.189107

**Published:** 2026-04-22

**Authors:** Rithika Behera, Yuechen Zhou, Peter H. Gerges, Jingyu Fan, Tracy Tabib, Alyxzandria M. Gaydosik, Mengqi Huang, Jishnu Das, Elena Pachera, Amela Hukara, Ying Tang, Florian Renoux, Miranda Tai, Oliver Distler, Gabriela Kania, Stephen Y. Chan, Harinder Singh, Eleanor Valenzi, Robert Lafyatis

**Affiliations:** 1 Division of Rheumatology and Clinical Immunology and; 2 Center for Systems Immunology and Department of Immunology, University of Pittsburgh, Pittsburgh, Pennsylvania, USA.; 3 Center for Experimental Rheumatology, Department of Rheumatology, University Hospital Zurich, University of Zurich, Schlieren, Switzerland.; 4 Division of Cardiology, Cardiovascular Institute, and; 5 Division of Pulmonary, Allergy and Critical Care Medicine, University of Pittsburgh, Pittsburgh, Pennsylvania, USA.

**Keywords:** Pulmonology, Vascular biology, Endothelial cells

## Abstract

Systemic sclerosis (SSc) is characterized by fibrosis and vasculopathy affecting the skin and internal organs, leading to multiorgan dysfunction. Injury of microvascular endothelial cells (ECs) in SSc impairs blood flow and causes tissue ischemia, leading to vascular complications such as Raynaud’s, digital ulcers, and pulmonary hypertension (PH). PH in SSc presents as group 1 pulmonary arterial hypertension or as group 3 PH related to hypoxia and interstitial lung disease (ILD), both major causes of mortality. Analysis of multiome data from SSc ILD-PH lungs inferred transcription factors regulating EC phenotype, including FOSL2. Overexpression of FOSL2 in transgenic mice (*Fosl2^tg^*) leads to vascular changes mirroring human SSc-PH, such as intimal thickening and fibrosis. scRNA-Seq analysis of altered EC gene expression in *Fosl2^tg^* mice showed strong overlap with altered EC gene expression in SSc-ILD-PH. Overlapping as well as discrete EC gene expression in Sugen/hypoxia- and hypoxia-treated mice suggested that FOSL2 regulates both hypoxia-dependent and -independent pathways in *Fosl2^tg^* mice and SSc-ILD-PH. A deep learning model, ChromBPNet, inferred increased AP-1 binding at base pair resolution in SSc-ILD-PH ECs, and binding to the same motifs was found upon FOSL2 overexpression in primary vascular ECs, highlighting FOSL2’s key role in driving the pathological changes seen in SSc-ILD-PH.

## Abstract 2

Our study indicates that FOSL2 drives endothelial cell apoptosis, contributing to microvasculopathy and vascular remodeling. The Fosl2 transgenic mouse model offers insights into the disease mechanisms, showing that Fosl2, a member of the AP-1 transcription factor family, plays a pivotal role in SSc-ILD-PH.

## Introduction

Systemic sclerosis (SSc) is a rare autoimmune disorder characterized by vasculopathy and fibrosis of the skin and internal organs that leads to multiorgan dysfunction. The etiology of the disease remains unknown, and currently there are very limited therapeutic options. Microvascular endothelium regulates blood flow, maintaining a balance between coagulation and fibrinolysis, and transportation of nutrients. In SSc, changes in microvessels, initiated by damage and apoptosis of endothelial cells (ECs), is thought to trigger reduced blood flow and tissue ischemia, leading to vascular manifestations such as Raynaud’s phenomenon, digital ulcers, and pulmonary hypertension (PH) (1, 2). Prolonged damage to ECs in SSc can lead to loss of vessel elasticity, chronic tissue ischemia, and hypoxia, resulting in inflammation, organ dysfunction, and morbidity such as digital necrosis, scleroderma renal crisis, and progressive pulmonary hypertension (PH).

PH in SSc occurs in several settings. Some patients develop pulmonary arterial hypertension (PAH), most commonly associated with limited cutaneous disease, and grouped with group 1 PH. Other patients with SSc develop PH in the context of hypoxia and interstitial lung disease (ILD, group 3 PH), though the role of ILD and hypoxia in SSc pulmonary vasculopathy remains unknown. Although these patients are classified in different PH groups, vascular disease and fibrotic disease in SSc are closely linked, and the vascular disease may be intimately involved in driving the fibrotic disease and not just an associated complication of lung fibrosis. PH and ILD are the leading causes of death in patients with SSc (3) and up to 75%–90% of patients with SSc present with ILD of some degree (4). PH affects 31% of patients with significant SSc-ILD (5). The mortality rate due to ILD in patients with SSc is approximately 33%, and survival rates decrease when both pulmonary manifestations exist (6–8). In the majority of patients with SSc, vascular remodeling, inflammation, and parenchymal loss due to fibrosis contribute to PH (9); however, the precise pathophysiology and aberrant molecular mechanisms are not well understood.

The *Fosl2* transgenic mouse model (*Fosl2^tg^*) displays both fibrotic and vascular features of human SSc-ILD-PH. A study conducted by Maurer et al. (10) showed that transgenic mice overexpressing *Fosl2* exhibit significant vascular remodeling in their pulmonary arteries and display characteristics found in PH associated with SSc in humans such as intimal thickening with concentric laminar lesions, medial hypertrophy, inflammatory infiltrates surrounding blood vessels, and fibrosis in vessel walls. Their study also indicated that *Fosl2* may have a role in the onset of microvasculopathy by triggering EC apoptosis and diminishing both EC migration and chemotaxis (11). FOSL2 is a member of the activator protein-1 (AP-1) family of transcription factors (TFs). AP-1 is composed of heterodimers of JUN (JUNB*,* JUND, c-JUN) and Fos proteins (FRA-1, FRA-2/FOSL2, c-FOS, FOSB). These members are considered as immediate early genes and control a variety of responses such as apoptosis, wound healing, proliferation, and cancer. Fra-2/FOSL2 encoded by the *FOSL2* gene has been reported to be overexpressed in fibrotic lungs of patients with SSc (11–13). The interaction between the chromatin structure and TFs plays a vital role in controlling gene regulatory networks and the overall transcriptome of cells (14). This relationship influences how genes are expressed, which ultimately determines the state of the cell — whether it functions normally as a healthy cell or undergoes changes that lead to disease. By affecting these fundamental processes, the chromatin and TFs together help shape the cell’s identity and its responses to various internal and external signals. By directly analyzing changes in chromatin accessibility, we show here that *FOSL2* is predicted to regulate the altered state of ECs from patients with SSc-ILD and PH. Reexamining the EC states in mice overexpressing *Fosl2* indicates that this TF drives downstream gene expression seen in both human and murine PH, and when overexpressed, *Fosl2* binds to *cis*-elements, showing upregulated TF binding in human SSc-ILD-PH lungs.

## Results

### Single-cell multiome sequencing of SSc-ILD-PH and healthy control lungs can identify different subtypes of ECs.

To investigate the perturbed gene regulatory programs in SSc-ILD-PH, we performed single-cell multiome sequencing on explanted lung tissue from patients with SSc-ILD-PH (*n* = 9) and from healthy controls declined for transplantation (*n* = 6, Supplemental Table 1; supplemental material available online with this article; https://doi.org/10.1172/jci.insight.189107DS1). A total of 79,125 cells from SSc-ILD-PH and control lungs passed quality control (Supplemental Figure 1 and Supplemental Table 2) and were integrated using the Harmony (15) algorithm to remove the observed batch effects between samples (Supplemental Figure 2, A–C). We identified a total of 23 clusters (Supplemental Figure 2, D and E). Cell-type identities were assigned to each cluster based on expression of canonical marker genes (Supplemental Figure 2F and Supplemental Table 3).

**Figure 1:**
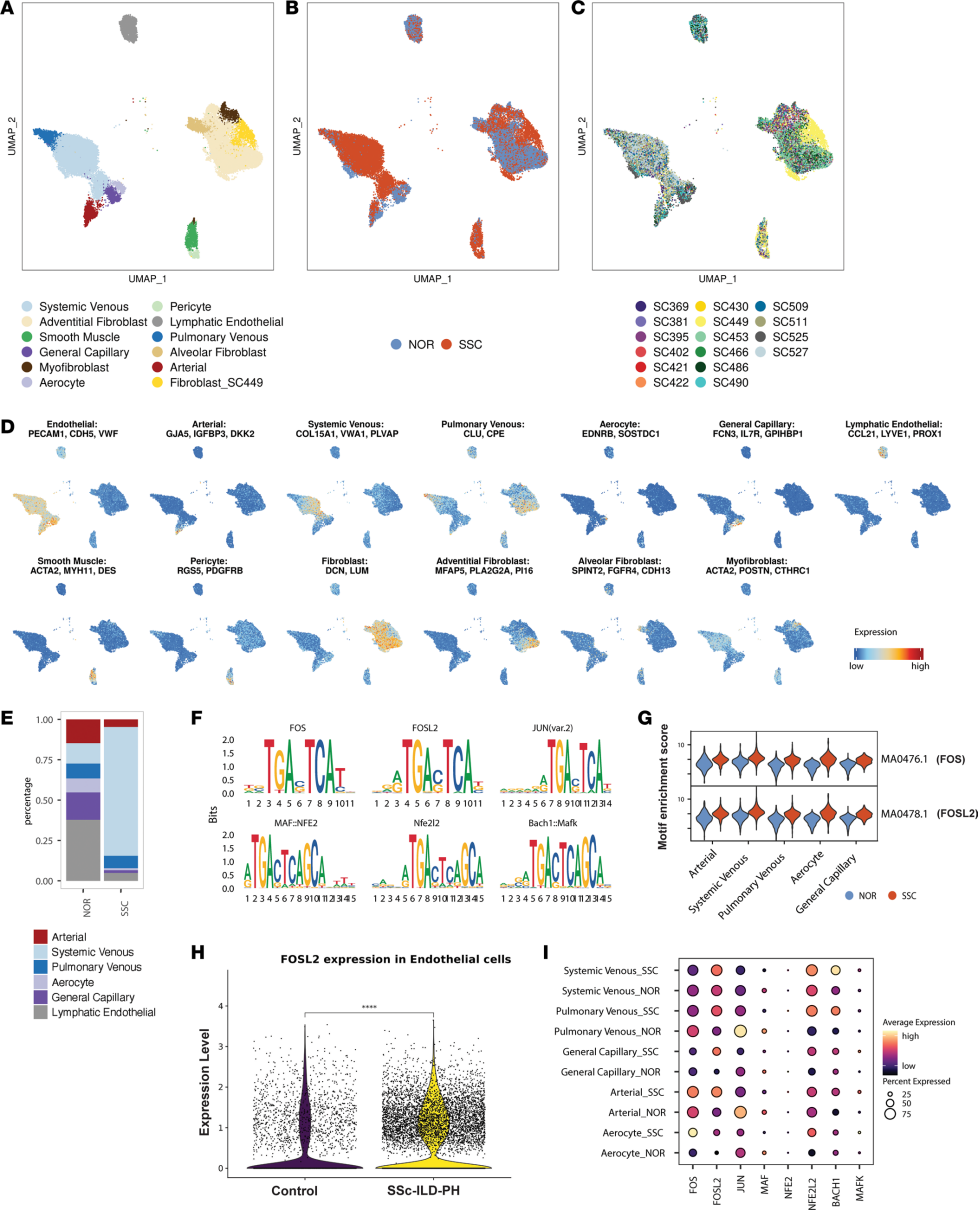
Single-cell multiome sequencing showed enrichment of FOS and FOSL2 motifs in SSc-ILD-PH vascular ECs. (**A**–**C**) UMAP visualization of endothelial and mesenchymal populations in SSc and control (NOR) lung tissues. Cells are colored by (**A**) cell types, (**B**) health status, or (**C**) samples. (**D**) Expression patterns of gene markers used for identification of endothelial or mesenchymal subpopulations. (**E**) Bar plot shows the percentage of each endothelial or mesenchymal subpopulation from control (NOR) and SSc-ILD-PH lungs. (**F**) Top 6 motifs showing enrichment in SSc-ILD-PH ECs compared with control ECs. (**G**) Violin plots showing chromVAR enrichment scores of MA0476.1 (FOS) and MA0478.1 (FOSL2) in each endothelial subpopulation from control (NOR; blue) and SSc-ILD-PH (red) lungs. (**H**) FOSL2 gene expression in ECs from lungs of healthy controls and patients with SSc-ILD-PH. (**I**) Average expression (dot color) of selected AP1 family transcription factors and percentage of cells showing expression (dot size) in endothelial subpopulations from healthy controls and patients with SSc-ILD-PH.

To better understand the transcriptional regulatory mechanisms associated with SSc-ILD-PH vasculopathy, we subclustered the endothelial and mesenchymal populations (Figure 1, A–C, and Supplemental Figure 2G). Based on literature gene markers (16), we identified 6 endothelial subpopulations including *GJA5*^+^*IGFBP3*^+^*DKK2*^+^ arterial ECs, *COL15A1*^+^*VWA1*^+^*PLVAP*^+^ systemic venous ECs, *EDNRB*^+^*SOSTDC1*^+^ aerocytes, and *FCN3*^+^*IL7R*^+^*GPIHBP1*^+^ general capillary ECs. Fibroblasts were annotated based on the expression of previously reported markers for adventitial (*MFAP5*, *PLA2G2A*, and *PI16*), alveolar (*SPINT2*, *FGFR4*, and *CDH13*), and myofibroblast (*ACTA2*, *POSTN*, *CTHRC1*) subtypes (17, 18) (Figure 1D and Supplemental Table 4). We additionally identified 1 fibroblast cluster primarily composed of cells from a single patient, which had minimal expression of previously identified gene markers for fibroblast subtypes. Notably, comparing SSc-ILD-PH with healthy control lung samples, we observed a marked increase in the percentage of systemic venous ECs and a decrease in the percentage of arterial ECs (Figure 1E).

### Gene regulatory network analysis of vascular ECs in SSc-ILD-PH and healthy control lungs shows an enrichment of FOSL2 regulatory network.

To identify TFs associated with SSc-ILD-PH disease pathogenesis in ECs, we performed motif enrichment analysis using chromVAR (19). We found enrichment of binding sites for AP-1 family proteins (FOS, FOSL2, JUN, MAF, and MAFK) and their binding partners (NF-E2 and NFE2L2) in SSc vascular ECs compared with healthy control vascular ECs (Figure 1F and Supplemental Table 5). Notably, FOS and FOSL2 motifs were ranked as the top 2 most differentially enriched motifs, both showing higher activity across all vascular EC subtypes in SSc-ILD-PH compared with healthy control lungs (Figure 1G). On comparing the differential gene expression in healthy control and SSc-ILD-PH lungs, we found that expression of *FOSL2* was upregulated in ECs from SSc-ILD-PH lungs compared with healthy control lungs (Figure 1H). Notably, *FOSL2* was upregulated in all the vascular EC subtypes from SSc-ILD-PH lungs (Figure 1I). *FOS* also showed modestly increased expression restricted to arterial ECs and aerocytes, as did *NFE2L2* and *BACH1*.

**Figure 2:**
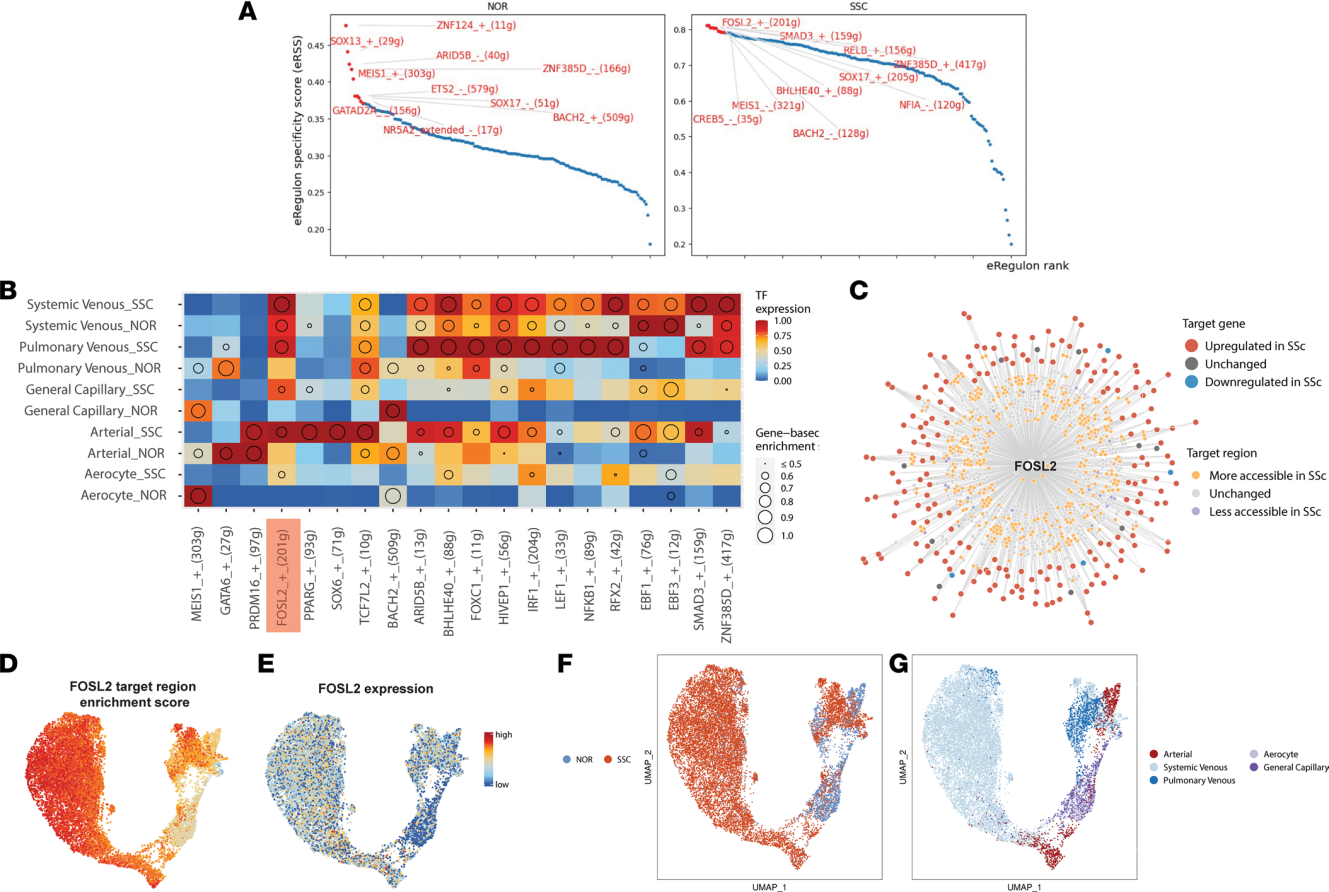
SCENIC^+^-identified enrichment of FOSL2 eRegulons in SSc-ILD-PH vascular EC populations. (**A**) TFs ranked according to eRegulon specificity score. TFs colored in red show the top 10 TFs for each condition. (**B**) Visualization of the FOSL2 eRegulon generated using SCENIC^+^. Predicted target regions and genes are color-coded by differential accessibility or expression (*P*_adj_ < 0.05) in SSc-ILD-PH ECs compared with controls. (**C**) TF expression and gene-based enrichment scores (dot size) for high-quality activator eRegulons. (**D** and **E**) Dimensionality reduction of vascular ECs using eRegulon enrichment scores. Cells are colored by (**D**) cell type or (**E**) health status. (**F**) Patterns of region-based enrichment score of FOSL2 eRegulon in the UMAP visualization of ECs. (**G**) FOSL2 expression patterns in the UMAP visualization of ECs.

We took advantage of joint chromatin accessibility and gene expression profiling to explore potential genomic regions and downstream genes targeted by disease-associated TFs using SCENIC^+^ (20). We identified 161 unique enhancer-driven regulons (eRegulons) targeting a total of 32,463 regions and 7,122 genes. We focused on high-quality eRegulons showing robust correlations between TF expression and target region enrichment score (correlation coefficient > 0.5 or < –0.5, Supplemental Figure 3A). A total of 20 activator eRegulons (positive TF-gene correlation, Figure 2A and Supplemental Figure 3B) and 5 repressor eRegulons (negative TF-gene correlation, Supplemental Figure 3, C and D) met these criteria.

Clustering of vascular ECs by eRegulon target region enrichment score suggested cell type–specific and disease-specific effects on the gene regulatory networks (GRNs) (Figure 2, D and E). SSc ECs clustered mainly in one group that included both systemic venous cells and a second cluster composed mainly of pulmonary venous cells (Figure 2F). eRegulons for aerocytes and capillary ECs largely overlapped when comparing SSc-ILD-PH with healthy controls (Figure 2G). Most striking was the difference in eRegulon clustering with SSc-ILD-PH compared with control arterial ECs (Figure 2, F and G).

Consistent with our chromVAR analysis, *FOSL2* showed higher expression and enrichment of predicted target genes and regions in SSc-ILD-PH across all vascular EC subpopulations (Figure 2, A, B, F, and G). Notably, among the 862 regions in the *FOSL2* eRegulon across all the EC subpopulations, 380 were significantly more accessible in SSc vascular ECs compared with healthy control vascular ECs, with only 22 showing decreased accessibility (Supplemental Table 6). Similarly, 187 out of predicted 201 *FOSL2* target genes showed significant upregulation in SSc vascular ECs, and only 4 were downregulated (*P*_adj_ < 0.05, Figure 2C).

**Figure 3:**
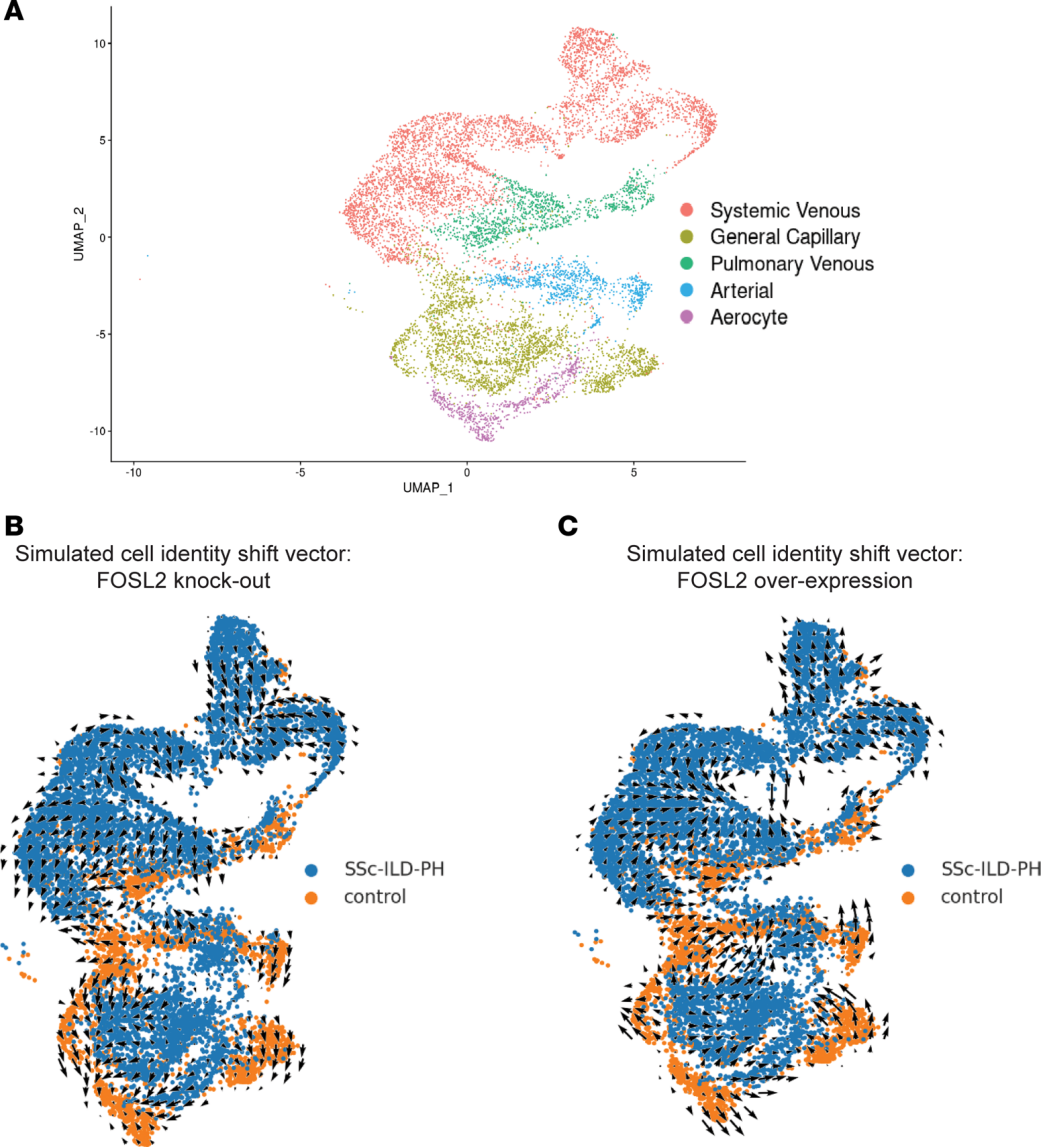
In silico FOSL2 perturbations using CellOracle identifies its role in maintaining healthy cell identity in vascular ECs. (**A**) UMAP dimensional reduction on ECs and its subtypes from lungs of controls (*n* = 16) and patients with SSc-ILD-PH (*n* = 16). Cells are colored by cell type. (**B** and **C**) Simulation of cell identity transitions due to FOSL2 perturbations: KO (**B**) and overexpression (**C**).

To predict the role of *FOSL2* in regulating cell identity across the healthy to diseased state, we applied CellOracle (21) to perturb *FOSL2* across vascular ECs from healthy and SSc-ILD-PH lungs in silico. We created a GRN for both the healthy and SSc vascular ECs using the single-cell ATAC-Seq data. This network identified TF-binding motifs (including *FOSL2*) in our dataset. We performed in silico KO (gene expression set to 0) and overexpression (gene expression increased to 1.5 times the highest expression) perturbations of *FOSL2* in 11,720 vascular ECs. Clustering by gene expression recapitulated vascular EC subpopulations (Figure 3A). Our results based on simulation and vector visualization on the GRN model indicate a shift in vascular ECs from a diseased to healthy state when *FOSL2* is knocked out (Figure 3B) and an opposite trend, from a healthy state to diseased state when *FOSL2* is overexpressed (Figure 3C). These results collectively suggest a role for *FOSL2* in driving transcriptional and epigenetic alterations in SSc-ILD-PH vascular ECs.

### Single-cell RNA-Seq identifies cellular and transcriptomic changes in ECs from Fosl2^tg^ mice.

**Figure 4:**
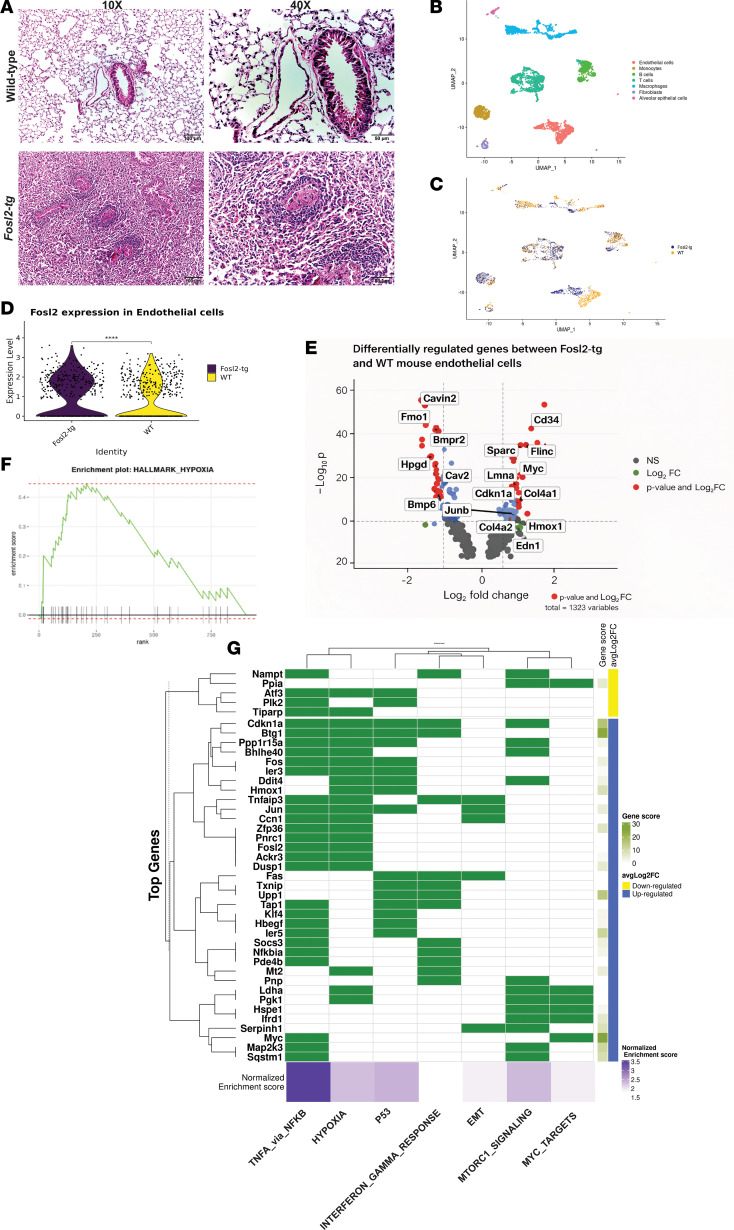
Vascular ECs from Fosl2^tg^ mice have dysregulated gene expression. (**A**) H&E staining on lungs isolated from WT and Fosl2^tg^ mice. Scale bars: 50 μm and 100 μm. (**B** and **C**) UMAP dimensional reduction visualization of cells isolated from single-cell RNA-Seq of WT and Fosl2^tg^ mice lungs. Cells are colored by (**B**) cell type or (**C**) mouse origin. (**D**) Fosl2 expression in ECs from lungs of WT and Fosl2^tg^ mice. (**E**) Differentially expressed genes between ECs from Fosl2^tg^ and WT mice. (**F**) Enrichment plot generated of Hallmark hypoxia. (**G**) Top differentially expressed genes from lungs of WT and Fosl2^tg^ mice that are a part of the top Hallmark gene sets by GSEA.

To further explore the function of *FOSL2* in vitro, we studied *Fosl2* transgenic mice (*Fos^tg^*) in which the murine *Fosl2* gene is expressed under the control of the MHC1 promoter H2Kb (12, 22). The histology on lung sections from WT mice showed well-formed air spaces, bronchioles, vascular channels, and thin-walled venules (Figure 4A). *Fosl2^tg^* mice lungs showed loss of air space and dense infiltration of fibrotic and inflammatory cells, as well as vascular smooth muscle cell hypertrophy, inflammatory vascular cuffing, and obliteration of vascular lumen (Figure 4A).

To explore changes in molecular characteristics at the single-cell level, we carried out single-cell RNA (scRNA) sequencing on lung tissue samples from 3 WT mice and 3 *Fosl2^tg^* mice. To minimize batch effects, all 6 samples were processed simultaneously using unique indexing and library preparation. We examined a total of 4,460 cells from 6 mice lung samples (Supplemental Table 7 and Supplemental Figure 4B) and identified 7 distinct cell types based on gene expression markers widely used to identify different mouse cell populations (Figure 4B). Cell type–specific markers for each cluster were identified based on the genes with the highest variable expression relative to all the other clusters (Supplemental Figure 4A and Supplemental Table 8). *Vwf*, *Lyve1*, *Pecam1*, and *Tek* expression identified a discrete cluster of cells and provided a strong transcriptome signature for murine vascular ECs (Supplemental Figure 4E).

On comparing the proportions of each cell type in WT and *Fosl2^tg^* mice, there was a decrease in the number of ECs, B cells, and macrophages and an increase in the cell proportions of T cell and monocyte-like macrophages identified from *Fosl2^tg^* mice (Supplemental Figure 4C). Cells in most of the populations showed similar transcriptomic changes when comparing WT with *Fosl2^tg^* mice, except for the myeloid and EC compartments, which showed divergent clustering on uniform manifold approximation and projection (UMAP) plots (Figure 4C). *Fosl2* gene expression was found to be increased in the vascular ECs from *Fosl2^tg^* mice (Figure 4D). *Fosl2* expression was slightly lower in T cells but not significantly different in other cell populations.

To better understand the heterogeneity and learn more about the different subpopulations, we subclustered the ECs. However, we couldn’t identify any markers to differentiate between the different subpopulations of ECs. To understand the disease-associated changes in vascular ECs due to *Fosl2* overexpression, we compared the differential gene expression between *Fosl2^tg^* and WT mice. Several of the upregulated genes in *Fosl2^tg^* mice have been reported to be increased in PH and are markers of disease progression, such as *Col4a1*, *Col4a2*, and *Edn1* (23) (Figure 4E). Other genes differentially overexpressed in *Fosl2^tg^* mice are involved in vascular remodeling and hypoxia, such as *Sparc*, *Rgcc*, *Anxa2*, and *Abl2* (24–27) (Figure 4E and Supplemental Table 9). We also found *Bmp6* and *Bmpr2* gene expression to be downregulated in *Fosl2^tg^* mice compared with WT mice. We used the Hallmark gene sets from Gene Set Enrichment Analysis (GSEA) to find enriched pathways from the differentially expressed genes in ECs from *Fosl2^tg^* compared with WT mice. Of all the Hallmark gene sets, TNF-α signaling through NF-κB, P53, MTORC1, and hypoxia had the highest normalized enrichment scores (*P*_adj_ < 0.05) (Figure 4, F and G and Supplemental Figure 4, D and G).

**Figure 5:**
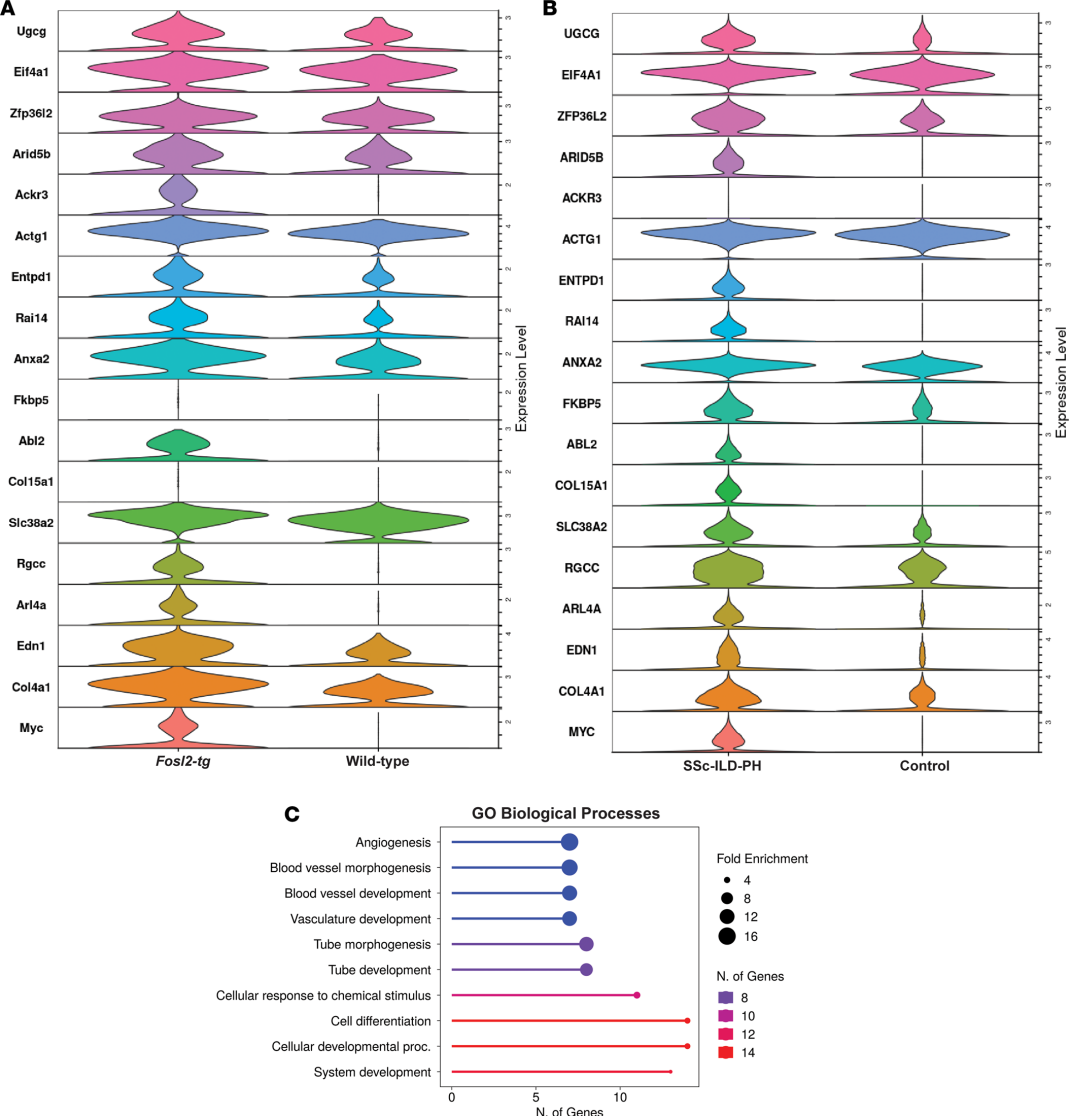
Transcriptomic similarity in ECs from Fosl2^tg^ mice and patients with SSc-ILD-PH. (**A** and **B**) Gene expression in ECs from Fosl2^tg^ mice (**A**) and patients with SSc-ILD-PH (**B**). (**C**) GO biological processes associated with the 18-gene overlap in Fosl2^tg^ mouse and SSc-ILD-PH lung ECs.

We compared results from the mouse data with human data to identify similarities to human vascular ECs isolated from the lungs of patients with SSc-ILD-PH. We used an scRNA-Seq dataset comprising lungs from patients with SSc-ILD-PH (*n* = 16) and healthy controls declined for transplantation (*n* = 15) (28). We used this dataset because it is larger than the single-cell multiome dataset, providing increased statistical power. The scRNA-Seq dataset identified identical vascular EC subpopulations as in the multiome dataset (28). As before (Figure 1H and Figure 2G), we observed increased *FOSL2* gene expression in the vascular ECs from patients with SSc-ILD-PH compared with healthy controls. We identified 295 genes to be differentially upregulated in vascular ECs from SSc-ILD-PH lungs, and 320 genes were differentially upregulated in vascular ECs from *Fosl2^tg^* mice (average log_2_ fold-change [FC] ≥ 0.32 and *P*_adj_ < 0.05; Supplemental Figure 4F). In addition,18 genes were differentially upregulated in ECs from both *Fosl2^tg^* mice and patients with SSc-ILD-PH (*P* < 0.05, Figure 5, A and B, and Supplemental Figure 4F). Genes such as *ANXA2* are sensitive to oxygen changes (29), and *MYC* and *ANXA2* have been reported to be increased during hypoxia in PH (30) and to induce angiogenesis (31). These observed common genes support similarity in human and murine disease. Gene ontology (GO) biological process analysis on these 18 common genes showed enrichment of angiogenesis, blood vessel formation, and tube development (Figure 5C).

### FOSL2 regulation of EC and T cell phenotypes before onset of significant fibrosis.

**Figure 6:**
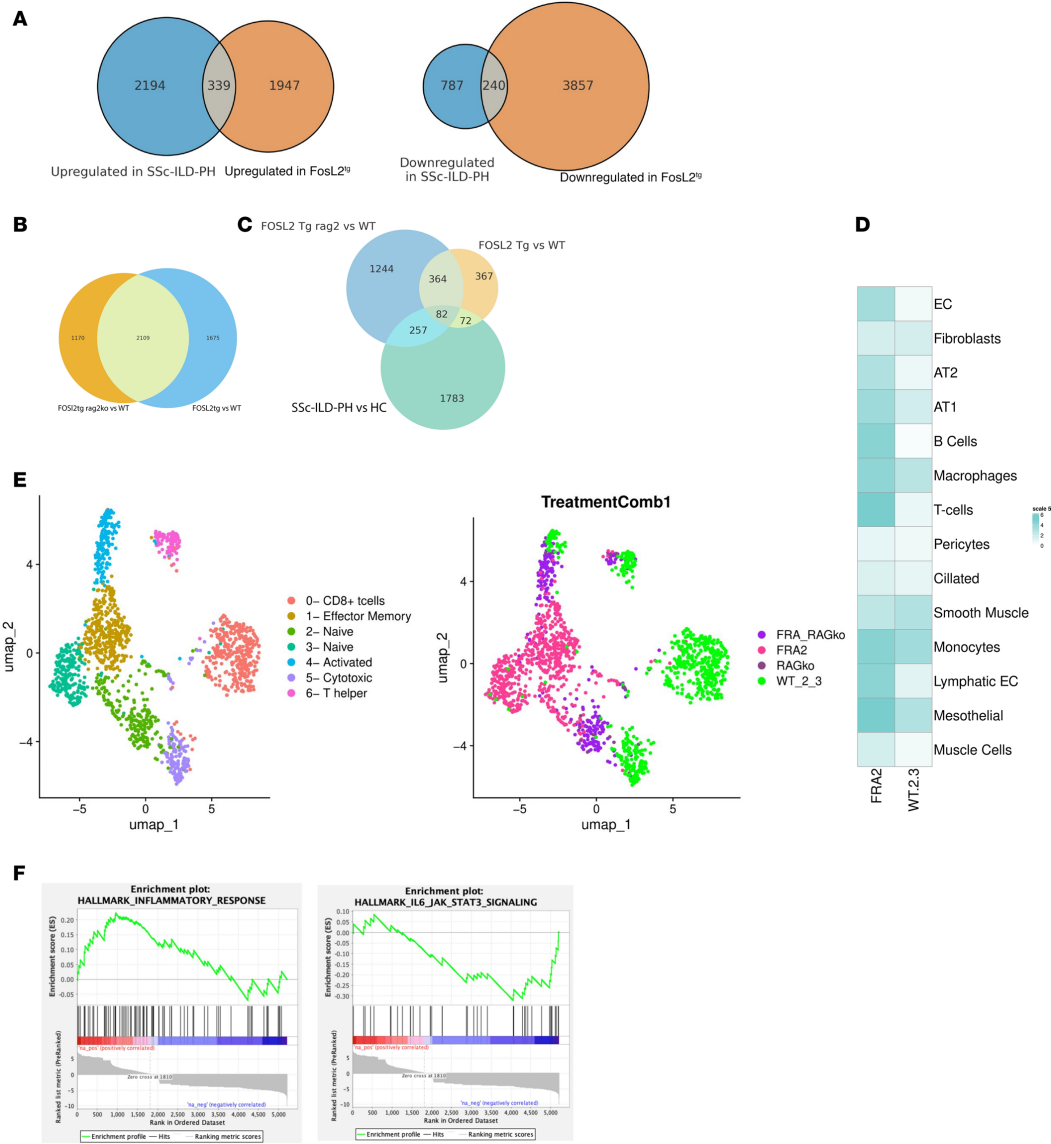
Altered gene regulation in Fra2^tg^ and SSc-ILD ECs. (**A**) Overlapping genes (*P*_adj_ < 0.05 and log_2_FC > 0.25) upregulated (hypergeometric *P* value: 6.91 × 10^–76^) and downregulated (hypergeometric *P* value: 1.54 × 10^–55^) when comparing Fra2^tg^ with WT mice and SSc-ILD-PH versus HC ECs. (**B**) Overlapping upregulated and downregulated genes (*P*_adj_ < 0.05 and log_2_FC > 0.1) in ECs when comparing Fra2^tg^ with WT and Fra2^tg^ rag2-KO versus WT mice (hypergeometric *P* value: 1.55 × 10^–268^). (**C**) Overlap of upregulated EC genes (*P*_adj_ < 0.05 and log_2_FC > 0.25) when comparing FOSL2^tg^ with WT, FOSL2^tg^ rag2-KO with WT, and SSc-ILD with HC lungs; Monte Carlo overlap *P* = 5.0 × 10^–5^. (**D**) Fosl2 expression in different cell populations in Fosl2^tg^ and WT mice. (**E**) Subclustering of T cells from Fosl2^tg^ mice. (**F**) GSEA pathway analysis of genes regulated in Fosl2 lung Treg cells.

To more clearly separate fibrosis and inflammation and to better understand whether the effect of FOSL2 overexpression on ECs in murine lung disease is direct or indirect (related to hypoxia or inflammation), we next examined scRNA-Seq gene expression in *Fosl2^tg^* mice (*n* = 2) before the onset of significant lung fibrosis compared with WT mice (*n* = 2) and in *Fosl2^tg^* mice bred to *rag2*-KO mice (*Fosl2^tg^rag2ko*, *n* = 2), along with *rag2*-KO mice (*rag2ko,* *n* = 1). In these *Fosl2^tg^* mice, perivascular inflammation and smooth muscular hyperplasia was apparent but fibrotic disease was minimal by H&E (Supplemental Figure 5) and trichome stains (data not shown). scRNA-Seq was carried out on paraffin embedded sections using FLEX technology (10x Genomics) (32). Sequence reads were mapped and a total of 40,209 cells included from the 7 samples. Cells were clustered, and cell populations were identified by marker genes using Azimuth and manual assignment (Supplemental Figure 6A and Supplemental Table 10 shows cell counts/cluster). Genes upregulated and downregulated in ECs from these mice overlapped largely with regulated genes in the more fibrotic lungs (data not shown). This FLEX RNA-Seq analysis showed more ECs, enabling arterial, capillary, and venous ECs to be discretely separated (Supplemental Figure 6B). Gene expression was generally consistent among phenotypes (Supplemental Figure 6C) and across samples (Supplemental Figure 6D). The change in arterial EC gene expression in *Fosl2^tg^* mice compared with WT mice largely overlapped with the changes in gene expression of ECs found in the other clusters (data not shown). Since lungs contain relatively few arterial cells (Supplemental Figure 6B, cluster 4), we combined all ECs to provide more robust comparisons of statistically significantly regulated genes in *Fosl2^tg^* mice. *Fosl2^tg^* compared with WT mouse EC shared 339 upregulated and 240 downregulated genes with SSc-ILD-PH compared with HC lung ECs (*P*_adj_ < 0.05 and log_2_FC > 0.25 for each comparison, Figure 6A and Supplemental Table 11). GO implicated multiple pathways in the overlapping upregulated genes, notably including positive regulation of leukocyte cell-cell adhesion (GO:1903039), regulation of TOR signaling (GO:0032006), cellular response to TGF-β stimulus (GO:0071560), and multiple pathways associated with RNA metabolism (Supplemental Table 12).

We next examined the role of the immune system in affecting the EC phenotype. *Rag2ko* mice showed few differences in EC gene expression compared with WT ECs (data not shown). To show the set of genes consistently upregulated by *Fosl2* that are not dependent on B cells and T cells, we compared *Fosl2^tg^* with *Fosl2^tg^rag2ko* ECs. Genes upregulated in *Fosl2^tg^* compared with WT overlapped strongly with genes upregulated in *Fosl2^tg^rag2ko* mice (Figure 6B), indicating that B cells and T cells are not requisite for most of the altered EC gene expression induced in *Fosl2^tg^* mice.

We further defined the upregulated genes shared between *Fosl2^tg^* and *Fosl2^tg^rag2ko* mice that overlap with genes upregulated in ECs from SSc-ILD-PH compared with healthy control lungs (*P*_adj_ < 0.05 and log_2_FC > 0.25, Figure 6C). This provided a group of 82 genes by the significant overlap between their overexpression in ECs in *Fosl2^tg^* and *Fosl2^tg^rag2ko* mice, and in SSc-ILD-PH (*P* = 5.0 × 10^–5^), including EDN1, SMAD5, JAG2, PDGFD, ABL2, as well as FOLSL2 (Supplemental Table 13A). Their upregulated expression in both overlapping gene sets suggests that their regulation is dependent on EC *Fosl2* overexpression but not on B cells and T cells.

To further address the role of Tregs, we reclustered the T cells in the scRNA-Seq data from nonfibrotic *Fosl2^tg^* mice (Figure 6E). The Tregs did not separate out as a discrete subcluster, and only a few cells (12 from WT and 10 from Fosl2^tg^ mice) expressed high levels of FOXP3, identifying them as Tregs. Surprisingly, these cells coexpressed CD8 rather than CD4, suggesting that they are CD8^+^ Tregs (33). The small number of cells did not provide significant corrected *P* value differences in gene expression when comparing *Fosl2^tg^* with WT mice. However, when comparing the *Fosl2^tg^* with WT Treg transcriptomes, GSEA showed upregulated “Hallmark _inflammatory_response” (nominal *P* value 0.039) and downregulated “Hallmark IL6_JAK_STAT3 signaling” (nominal *P* value 0.036). *Fosl2^tg^* Tregs showed upregulated but not statistically significant changes in markers of Treg activation: *Il10* (5.9-fold increase), *Havcr2* (i.e., Tim-3, 38.5-fold increase); *Tnfrsf18* (i.e., GITR, 3.5-fold increase) as well as *Gzmb*. *Foxp3* itself was increased 3.2-fold in *Fosl2tg* CD8-expressing Tregs (nominal *P* value = 0.00025). GO reinforced the GSEA, showing significant FDR-corrected “Regulation of lymphocyte differentiation (GO:0045619)” and related pathways (Supplemental Table 13B).

We then examined changes in T cells broadly, providing a more robust statistical comparison. A broad shift in Th1, Th2, and CD8 phenotypes was seen in the UMAP clustering (Figure 6E). Using GO to examine genes upregulated by all *Fosl2tg* T cells (FC > 2 and *P* value < 0.05) indicated multiple significant pathways (Supplemental Table 14), including “regulation of lymphocyte activation (GO:0051249).” Amongst the 13 genes in this pathway, *Fosl2^tg^* T cells showed increased expression of *Il5*, *Il21*, *Il13*, and *Il10*. Upregulated *Il5* and *Il13* suggest an activated Th2 response, *Il21* suggests an activated Tfh or Th17 response, and *Il10* suggests a regulatory response. Together, these results are consistent with activation of multiple T cell subsets in *Fosl2^tg^* mice and suggest that deficiency of Tregs may be only part of the T cell response driving inflammation and eventual fibrosis. *Fosl2^tg^* mice showed increased *Fosl2* expression in most cell populations, including T cells (Figure 6D).

### AP-1 inhibitor treatment of Sugen-hypoxia.

**Figure 7:**
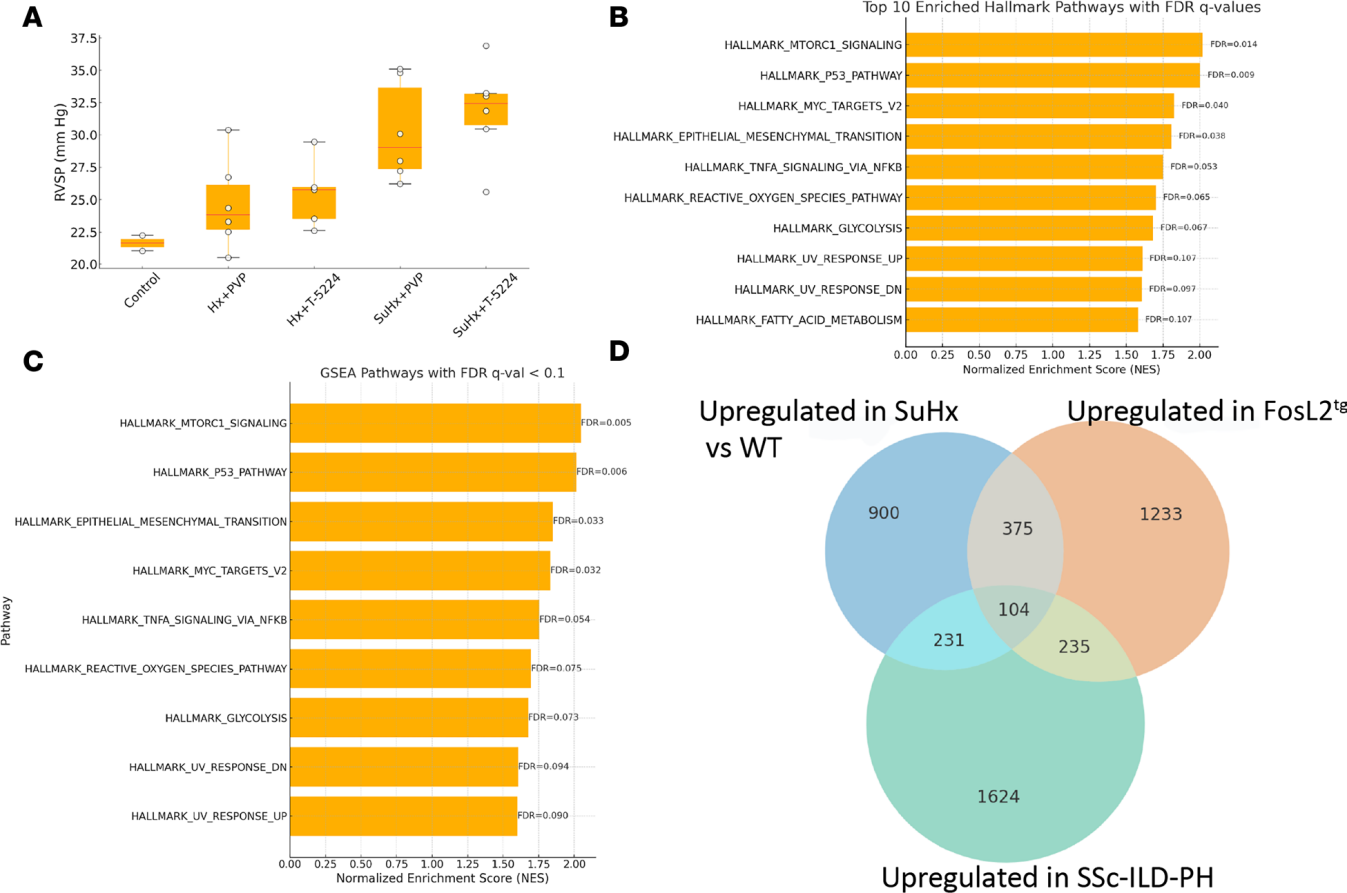
Ap-1 inhibitor T-5224 treatment of murine SuHx. (**A**) Pulmonary ventricular systolic pressure (PVSP) across experimental groups. Box plots show the distribution of PVSP values. Boxes indicate the IQR, with the horizontal line representing the median. Whiskers denote the range excluding outliers. (**B**) Pathways (FDRs as indicated) implicated in SuHx by GSEA of differentially regulated genes comparing SuHx with WT ECs (*P*_adj_ < 0.05). (**C**) Pathways downregulated by T-5224 in SuHx (FDRs as indicated). GSEA of differentially regulated genes comparing SuHx T-5224 with SuHx ECs (*P*_adj_ < 0.05). (**D**) Genes showing overlapping upregulated expression in Fosl2tg, SuHx, and SSc-ILD ECs.

We then tested the potential for broad AP-1 inhibition to ameliorate PH by treating mice with T-5224 during Sugen-hypoxia–induced (SuHx-induced) PAH. Mice (*n* = 12/group) were placed under hypoxia or placed under hypoxia after treatment with Sugen (SU5416, 20 mg/kg). These groups were split and treated daily by oral gavage with T-5224 (30 mg/kg; *n* = 6) dissolved in 10% polyvinylpyrrolidone (PVP) or 10% PVP (control; *n* = 6). At the end of the 21-day treatment period, the mice showed increased pulmonary ventricular systolic pressure under hypoxia (Hx) and further increased pressure with SuHx, as assessed by catheterization (Figure 7A). However, we did not observe a decrease in pulmonary ventricular systolic pressure associated with T-5224 treatment in either setting (Figure 7A). Further, T-5224 was not associated with any change in right ventricular/left ventricular plus septal mass (data not shown).

To further understand at a molecular level the effect of T-5224 on PAH, we examined EC gene expression by scRNA-Seq. A total of 45,153 cells from WT (*n* = 2), HxPVP (*n* = 1), HxT-5224, HxSuPVP (*n* = 2), and HxSuT-5224 (*n* = 1) mice were sequenced and clustered, and the cell clusters were identified as above (cell counts/cluster provided in Supplemental Table 10). SuHx profoundly altered EC gene expression, showing upregulated expression of 1,610/8,354 detected genes (*P*_adj_ < 0.05 and log_2_FC > 0.25). GSEA indicated that SuHx increased MTORC1, P53, Myc targets, epithelial-mesenchymal transition, and other GSEA Hallmark pathways (Figure 7B). In contrast, using the same criteria, only 55 genes upregulated in SuHx mice were inhibited in SuHx mice treated with T-5224. Although this suggested that T-5224 had a relatively modest effect on EC gene expression, the pathways affected by T-5224 strongly overlapped, downregulating the same 4 top pathways upregulated by SuHx (Figure 7C). These observations suggest that T-5224 partially reverses the molecular changes in ECs induced by SuHx.

### Overlapping EC genes between Fosl2^tg^ and SuHx.

Since we would anticipate that the SuHx model might be less dependent on AP-1 than the *Fosl2^tg^* model, we examined the relationship between the SuHx and *Fosl2^tg^* models and compared both with SSc-ILD-PAH. Upregulated genes in ECs from SuHx mice (*P*_adj_ < 0.05 and log_2_FC > 0.25; 1,610 genes) largely overlapped 479 genes (hypergeometric *P* value: 2.36 × 10^–28^) upregulated in ECs from *Fosl2^tg^* mice (*P*_adj_ < 0.05 and log_2_FC > 0.25; 1,947 genes). However, there were also significant differences in gene expression comparing the 2 models (Figure 7D).

### Overlapping genes between Fosl2tg, SuHx, and SSc-ILD-PH.

Reasoning that genes consistently regulated in PAH models are more likely key to pathogenesis, we compared EC genes regulated in these 2 models with SSc-ILD-PAH. Both models showed strong, highly significant overlap with altered gene expression in SSc-ILD-PH versus healthy control lung ECs: 104 genes overlapped between the 2 models and SSc-ILD-PH (Monte Carlo simulation *P* = 0.0013, Supplemental Table 15). Both models in parallel with SSc-ILD-PH showed upregulated expression of *Edn1*, *Jam2*, *Jag2*, *Pdgfd*, and *Fosl2*. Even more genes in each model showed highly significant overlap with SSc-ILD-PH versus healthy controls (231 genes discretely upregulated in SuHx and 235 genes discretely upregulated in *Fosl2^tg^* mice compared with WT), suggesting significant differences between the way these models affect pulmonary vascular disease.

To minimize challenges related to multiple comparisons, we selected 4 genes upregulated in *Fosl2^tg^* SuHx mice and SSc-ILD-PH (Supplemental Table 15) based on their roles in vascular biology, *Edn1*, *Pdgfd*, *Jag2,* and *Jam2*, and examined the relationship between their expression and the pretransplant mean pulmonary artery pressure of the lung explants. Of these genes, only *Jag2* showed a trend toward correlating with the patient mean pulmonary artery pressure (*r* = 0.61, nominal *P* value = 0.08).

### Hypoxia-regulated genes differ from Fosl2^tg^-regulated EC genes.

To test the role of hypoxia in *Fosl2^tg^* mice, we compared EC gene expression in *Fosl2^tg^* lungs showing minimal fibrosis with mice treated with hypoxia. Prefibrotic *Fosl2^tg^* mice did not show a significant GSEA Hallmark hypoxia pathway or significant hypergeometric overlap in gene expression with Hx-treated mice, whereas Hx-treated mice did show the Hallmark hypoxic pathway (Supplemental Table 17). Collectively, these studies do not support hypoxia as the main driver of altered EC gene expression in *Fosl2^tg^* mice early in the disease. In addition, we did not see evidence in ChomeVAR or ChromBPNet for altered HIF1a binding (data not shown). However, a subset of genes regulated in SSc-ILD-PH strongly overlapped with EC genes regulated in Hx-treated mice (Supplemental Table 16, 37 upregulated and 35 downregulated genes, *P*_adj_ < 0.05, log_2_FC > |0.25|, hypergeometric *P* value: 3.2 × 10^–21^).

### Elucidating the epigenomic landscape of FOSL2-overexpressing primary ECs.

To evaluate the effects of *FOSL2* overexpression on primary vascular ECs, we isolated ECs from healthy control human lungs that were declined for transplantation (*n* = 2). To investigate the regulation of EC chromatin accessibility by *FOSL2*, we examined changes in lung ECs upon *AdV-FOSL2* infection using ATAC-Seq. We used ChromBPnet (34) to correct for Tn5 enzyme bias and predict DNA accessibility at base-pair resolution to gain granular insight into the *cis*-elements regulated by *FOSL2* overexpression at single base-pair resolution. We trained separate ChromBPnet models for bulk ATAC data generated from primary control and *AdV-FOSL2*–overexpressing ECs and pseudo-bulk single-cell ATAC data from healthy control and SSc-ILD-PH vascular ECs. Using the ChromBPnet models, we extracted a base-level contribution score of each nucleotide and implemented TF-MoDISco (35) to identify motifs and their associated seqlets (motifs with high absolute attribution scores).

**Figure 8:**
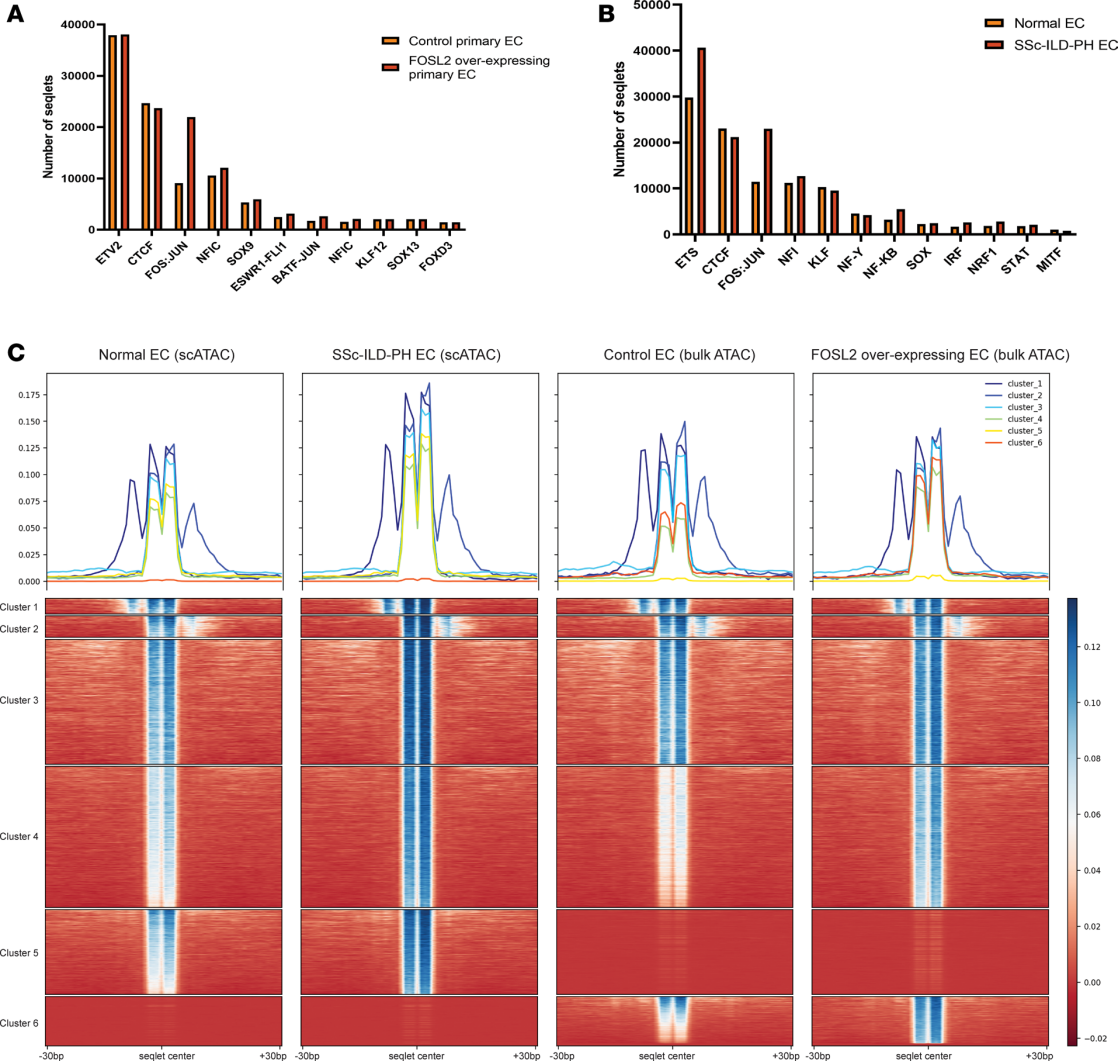
ChromBPNet identified FOS:JUN (AP-1) motifs to be expressed in SSc-ILD-PH and FOSL2 overexpressing ECs. (**A** and **B**) TF-MoDISco output on the number of seqlets identified from bulk ATAC data on primary control and FOSL2-overexpressing ECs (**A**), and single-cell ATAC data from healthy control and SSC-ILD-PH patient ECs (**B**). (**C**) Heatmap to observe contribution scores/genomic regions of AP-1 seqlets and nearby regions (± 30 base pair). Each row here is a specific genomic region with the AP-1 seqlet in the center. Each column is a contribution score associated with a particular nucleotide in the region. Cluster 4 has differences in contribution scores between single-cell ATAC healthy control and SSc-ILD-PH ECs, and primary control and FOSL2-overexpressing ECs, respectively.

**Figure 9:**
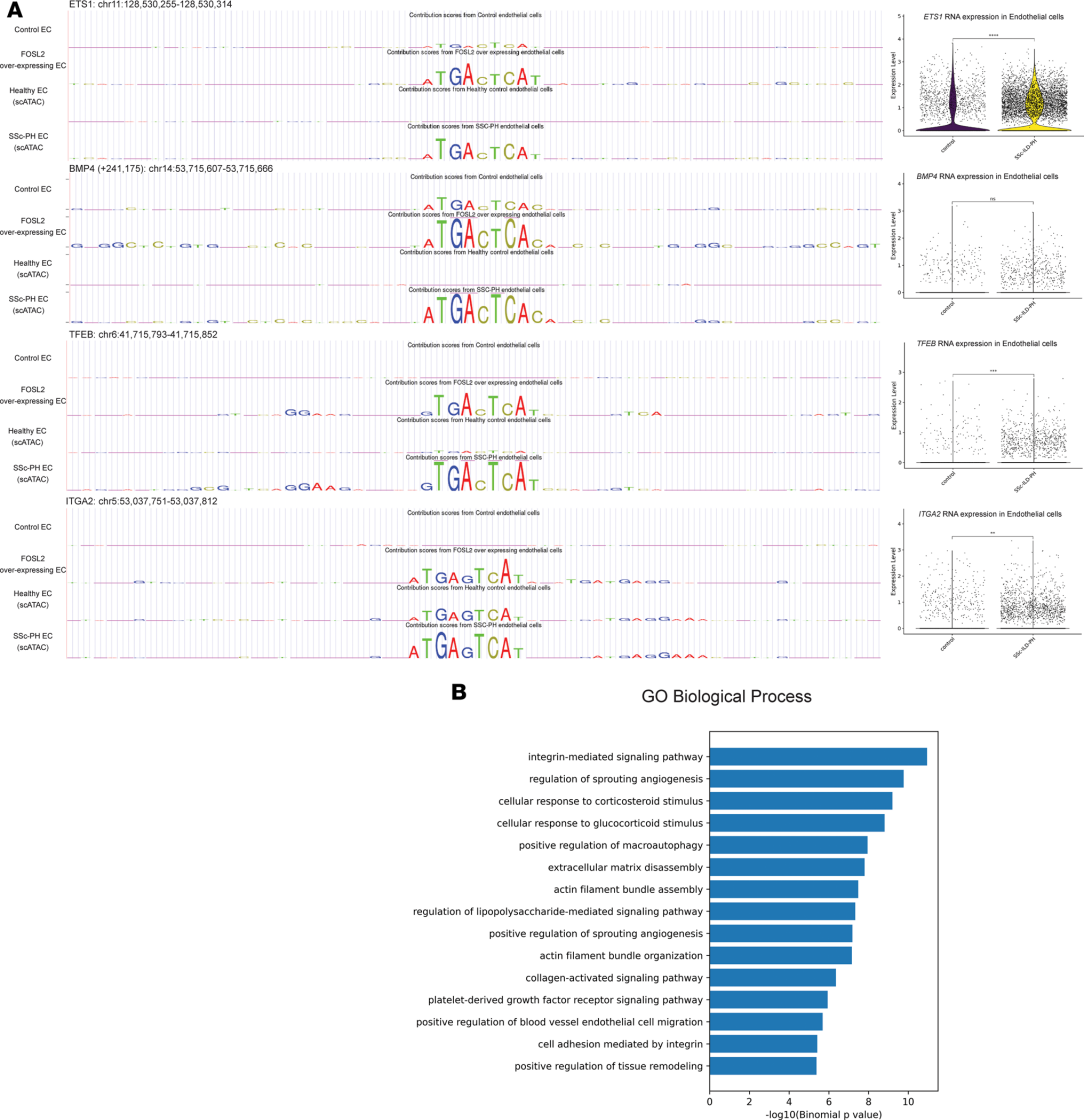
Parallel AP-1 binding in SSc-ILD-PH and FOSL2-overexpressing endothelial cells. (**A**) Genome browser visualization of FOS:JUN (AP-1) seqlets from genomic regions with highest difference in contribution scores from healthy control and SSc-ILD-PH ECs, and primary control and FOSL2-overexpressing ECs. RNA expression of nearest gene to the genomic region is indicated to the right. (**B**) GO biological processes associated with genomic regions in cluster 4.

TF-MoDISco identified the *FOS:JUN(AP-1)* motif as exhibiting an increased number of seqlets in *FOSL2*-overexpressing ECs and in SSc-ILD-PH vascular ECs (Figure 8, A and B). We then extracted all the *FOS:JUN(AP-1)* motif seqlets (TGAGTCA) and their respective contribution scores from our ATAC-Seq data and clustered them to find changes in contribution scores between primary control and *FOSL2*-overexpressing ECs and healthy control and SSc-ILD-PH vascular ECs (Figure 8C). Cluster 4 identified genomic regions with the same *FOS:JUN(AP-1)* motif showing increased contribution scores in both SSc-ILD-PH vascular ECs and *FOSL2*-overexpressing primary ECs. This enabled us to identify regions that have similar changes in AP-1 binding due to disease (SSc-ILD-PH) and *FOSL2* overexpression in ECs. Examining genomic regions that had the highest differences in their contribution scores between primary control and *FOSL2*-overexpressing ECs and healthy control and SSc-ILD-PH vascular ECs highlighted regions in the *ETS1*, *BMP4*, *TFEB*, and *ITGA2* genes (Figure 9A). SSc-ILD-PH ECs also showed upregulated expression of these genes (Figure 9A). The GO biological process associated with genes closest to *FOS:JUN(AP-1)* seqlets in cluster 4 pointed to integrin-mediated signaling pathway, regulation of angiogenesis, and extracellular matrix disassembly (Figure 9B).

In addition to these upregulated genes, we looked specifically at *BMPR2* due to its genetic association with familial PAH and its consistently downregulated expression in SSc-ILD-PH and murine models of PAH. We noted 2 adjacent consensus AP-1 motifs showing increased binding in the *BMPR2* first intron and increased binding upon *FOSL2* overexpression, suggesting that FOSL2 might downregulate *BMPR2* and other genes in certain contexts (Supplemental Figure 7).

## Discussion

The origin of and interconnections between vasculopathy, fibrosis, and inflammatory pathways in the lungs of patients with SSc-ILD-PH are not well understood. Our study indicates that *Fosl2* expression in ECs is a key driver in initiating and maintaining vascular pathology in patients with SSc-ILD-PH as well as *Fosl2^tg^* mice.

Machine learning through ChromBPnet provided single-nucleotide resolution of increased AP-1 binding. This provides strong evidence for the importance of these putative enhancers in the transition of healthy ECs to SSc-ILD-PH ECs. Three observations strongly infer that *FOSL2* is driving important aspects of this AP-1 activity and contributing to the altered vascular EC state in human SSc-ILD-PH. First, in silico analysis promotes this TF as both upregulated transcriptionally and associated with increased expression of predicted target genes in human SSc-ILD-PH ECs. Second, ECs from *Fosl2^tg^* mice show upregulated gene expression that parallels upregulated gene expression seen in human SSc-ILD-PH. These genes are mainly involved in blood vessel development and morphogenesis and have an active role in disease progression and vascular remodeling, one of the hallmarks of SSc-ILD-PH pathogenesis. Third, *FOSL2* overexpression in vitro leads to increased binding to most of the same AP-1 elements, showing increased AP-1 binding in single-cell multiome data from patients with SSc-ILD-PH.

It is likely that FOSL2/AP-1 does not act alone to regulate genes in SSc-ILD-PH ECs. Given that some of the seqlets showing increased binding in SSc-ILD-PH did not show increased binding with FOSL2 overexpression and vice versa, it appears that other AP-1–associated proteins and/or regulators of chromatin accessibility affect AP-1 binding in SSc-ILD-PH. *BACH2* shows downregulated expression in all the EC subpopulations and is predicted by SCENIC^+^ to affect the SSc-ILD-PH EC state. As it binds to the same AP-1 consensus sequence, *BACH2* might be affecting AP-1 binding at these sites. Expression of *FOS* is also upregulated in SSc-ILD-PH ECs and predicted to bind to the same consensus element. Although we cannot distinguish the relative importance of FOS versus FOSL2 in binding to these sites, our overexpression data indicate that increased *FOSL2* can lead to increased binding to these sites. In addition, the observed phenotype in *Fosl2* transgenic mice is distinct from the phenotype of *c-Fos* transgenic mice harboring a similar promoter. These mice develop osteosarcomas without reported ILD or PH (36).

SCENIC^+^ analysis infers other TFs that are potentially important in regulating the EC phenotype, such as PPARG, SOX6, and SMAD3. PPARG; SOX17, which binds to an overlapping consensus as SOX6; and SMAD3 have all been implicated in PH. ChromBPnet analysis also indicates increased binding of proteins to consensus elements for ETS family TFs, suggesting that these TFs are important in regulating the EC phenotype, as we recently described in ECs from SSc skin (37). As for AP-1, we have now clearly characterized the altered occupancy of these binding sites, leading the way for more precise identification of the specific TFs regulating the cell states.

Hallmark hypoxia genes were upregulated in ECs in the later fibrotic stage in *Fosl2^tg^* mice but not in the earlier stage of the disease. A significant subset of EC genes in Hx-treated mice were coregulated in and SSc-ILD-PH ECs, consistent with the classification of these patients into group 3 PH. However, even more genes were coregulated when comparing EC genes regulated at the early, prefibrotic stage of *Fosl2tg* with SSc-ILD-PH. In these mice, EC gene expression did not suggest hypoxia as a driving factor. Together these data support both hypoxia-dependent and hypoxia-independent regulation of the EC phenotype in SSc-ILD-PH. Several previous reports support the role of AP-1 in hypoxia. Hypoxia induces cell type–specific AP-1 activity through both JUN and FOS family members (38–40), and Chip-Seq data shows enhanced binding of FOSL2 and JUN in vitro in hypoxic human umbilical vein ECs (41). ATAC-Seq data from HeLa cells infers that hypoxia increases binding to HIF and AP-1 sites. However, the data presented here suggest that FOSL2 is also and perhaps more important in regulating hypoxia-independent pathways mediating EC dysfunction in PH.

Pathway analyses consistently implicated p53 and mTORC1 pathways in *Fosl2tg* and SuHx models and SSc-ILD-PH. p53 regulates apoptosis and controls cell survival and senescence (42). Wang et al. reported increased PAEC p53 in Hx and idiopathic pulmonary arterial hypertension, suggesting that it promotes EC apoptosis (43). However, another study has shown that EC deletion of p53 aggravates SuHx-induced PAH and regulates BMPR2 by inducing TLR3 and downstream IRF3 (44). Although neither p53 nor IRF3 binding was inferred in our SCENIC^+^ analysis, increased IRF1 was detected. Given that IRF1 and IRF3 binding sites are highly homologous, IRF1 eRegulon activity might represent IRF3. Both mTORC1 and mTORC2 have been studied in PAH, although are more strongly implicated in affecting vascular smooth muscle and mesenchyme (45, 46). mTORC1 inhibition with rapamycin alleviates the hypoxia-induced exacerbation of PAH in DP1-KO mice (45). In ECs, mTOR is important in maintaining EC integrity (47), but its role in ECs in PAH less studied. Recent studies have shown that *BMPR2* transcription is regulated by SIN3 as a coactivator and corepressor of gene expression (48). Although Sin3 has been shown to regulate AP-1 activity, there are no current reports directly implicating AP-1 in transcriptional regulation of BMPR2. On the other hand, loss of BMPR2 signaling has been associated with AP-1 activation (49), suggesting that altered BMP signaling might be affecting the inferred change in FOSL2 binding in SSc-ILD-PH.

Other studies show that AP-1 transcriptional regulation is important in vascular remodeling. Dominant negative c-Jun and AP-1 decoy oligonucleotides ameliorate balloon-induced intimal hyperplasia (50, 51). Although these studies focused on smooth muscle cells, decoy oligonucleotides were delivered to all layers of the injured artery, and dramatically increased AP-1 activity was shown by gel retardation (51). Although direct, specific FOSL2 inhibitors are not yet available, it can potentially be targeted by broad AP-1 inhibition as well as by epigenetic modulation. T-5224 blocks AP-1 binding to DNA, whereas ZN444B inhibits HDAC1 deacetylase activity on Sp1, disrupting Sp1 activation of the FOSL2 promoter (52). We did not detect an effect of T-5224, a broad AP-1 inhibitor, on pulmonary artery pressures but detected modest changes in gene expression in T-5224–treated mice, consistent with an inhibitory effect on the SuHx-induced EC phenotype. The lack of an observable effect on pulmonary pressures could be related to the relatively small sample size, insufficient inhibition of *Fosl2,* or coordinate inhibition of other AP-1 family members that might oppose the effect of *Fosl2* inhibition.

Previous studies in *Fosl2^tg^* mice have shown an activation of T cells and their contribution in systemic inflammation due to a reduction in Treg development (22). Few Tregs were detected in the scRNA-Seq study presented here, precluding statistically robust comparisons between *Fosl2tg* and WT mice. As previously reported, T cells in *Fosl2tg* mice show increased expression of Th2 as well as Th17 cytokines, suggesting broad T cell activation. However, altered EC gene expression in *Fosl2tg* mice was similar even in the absence of T and B cells, consistent with the notion that the effect of *Fosl2* on ECs does not depend on T and B cells. Rather the altered EC phenotype likely is at least partially responsible for the striking perivascular T cell accumulation in this model. In the *Fosl2^tg^* mouse strain used here, *Fosl2* expression was only modestly increased in pulmonary macrophages. However, we did see upregulated expression of *SPP1*/osteopontin and Arg in the monocyte/macrophage population, consistent with past observations (12). Although the relationship between fibrosis and vascular pathology remains uncertain in SSc-ILD-PH, collectively, our data suggest that altered EC gene expression may drive not only the vasculopathy but also contribute to the inflammation observed in *Fosl2^tg^* mice and SSc-ILD-PH lungs.

Our most exciting observations take advantage of the machine learning algorithm ChomBPNet. Although it clearly discovers important consensus binding elements de novo at base-pair resolution, it remains uncertain why HIF sites are not identified as showing increased binding in SSc-ILD-PH. Perhaps HIF is not triggered or only triggered transiently by the relatively preserved O^2^ tension in SSc-ILD-PH lungs compared with in vitro experimental systems. Chronic hypoxia in many studies shows differences from acute hypoxia where HIF proteins have been most studied. Although we cannot exclude some predisposition for ChromBPNet in its detection of binding, perhaps related to the affinity of the TF, HIF binding sites were also not detected as upregulated in chromVAR, which does not depend on this algorithm and looks for known binding elements. It does not appear to be an issue with the ability for ATAC-Seq to detect HIF binding elements, as ATAC-Seq has detected HIF binding motifs in hypoxic HeLa cells (53). Thus, our data strongly point to a more important role of AP-1 family and other TFs in SSc-ILD-PH.

In summary, our study shows remarkable alterations in binding to AP-1 *cis-*elements in ECs from patients with SSc-ILD-PH who have group 3 PH and strongly implicates *FOSL2* in the EC dysfunction that leads to pulmonary vascular disease in both *Fosl2tg* mice and in patients with SSc-ILD-PH.

## Methods

### Sex as a biological variable.

Our study examined male and female healthy control donors and SSc-ILD-PH lung samples. Similar findings were reported for both sexes, and sex was not considered as a biological variable.

### Tissue processing and cell culture.

Explanted lung tissues were finely minced and digested for 1 hour in a mix of 1 mg/mL Liberase DL (Roche, 05466202001) and DNase I (Sigma-Aldrich, D5025) at 37°C with occasional agitation. The digested tissue was filtered, centrifuged, and washed with 2% FBS in 1× PBS. Pulmonary microvascular ECs were isolated using the CD31 MicroBead kit (Miltenyi Biotec, 130-091-935) according to the manufacturer’s instructions and cultured in EGM-2MV (Lonza, CC-3202) with 1% antibiotic-antimycotic (Gibco, 15240062).

### Adenoviral infection.

Ready-to-use genetically modified recombinant adenovirus (DE1/E3) was purchased from Vector Biolabs. The *FOSL2* transgene overexpression was driven by a CMV promoter. Primary ECs were infected by viruses at MOI of 1,000 for 4 hours. Cell culture medium was then changed and cells were cultured for an additional 44 hours. The expression of *FOSL2* transgene in the transduced primary ECs was assayed using RNA-Seq and qPCR 48 hours after infection.

### Histology of mouse lungs.

Frozen mouse lungs were obtained in-house. WT (*Fosl-2^wt^*) littermates were used as controls. To generate *Fosl-2^tg^* mice, a vector containing the murine *Fosl-2* gene (exons 1 to 4, corresponding introns, and truncated untranslated regions under the control of the MHCI promotor H2Kb) were randomly inserted into the genome (54).

The lung tissue was fixed with 10% neutral buffered formalin for 48–72 hours and then paraffin embedded. Next, 5 μm thick sections were cut and baked at 65°C for 45 minutes to adhere the section. The sections were deparaffinized, rehydrated, and stained with H&E.

### scRNA-Seq processing and data analysis.

Lungs from 3 WT and 3 *Fosl2^tg^* mice were digested using the mouse muscle kit (Miltenyi Biotec, 130-098-205). Samples were processed with 10x Genomics Chromium Single Cell 3′ v1.3 reagents as per the manufacturer’s protocol. The libraries were sequenced using the Illumina NovaSeq 6000 platform. Raw data were demultiplexed using Cell Ranger’s mkfastq function and aligned to the mm10 reference genome. Downstream data processing was performed with the R package Seurat v4.1.1. Potential doublets were predicted by the R package DoubletFinder (55) v2.0.3 and removed. Cells expressing less than 200 genes or more than 15% mitochondrial counts were excluded from further analysis. Gene expression matrix was normalized and scaled using NormalizeData and ScaleD atafunctions in Seurat, followed by dimensionality reduction using principal component analysis (PCA). A K-nearest neighbor graph was constructed using FindNeighbors function in Seurat. Cells were clustered with the Louvain algorithm, as implemented in FindClusters function. Cluster-specific markers were generated using the FindAllMarkers function, and cell identities were assigned based on extensive literature search of genes widely used. Clusters with low UMI counts and no known cell type–specific markers were considered low quality and removed.

### Murine Su-hx.

Male C57BL/6J mice (The Jackson Laboratory, stock number 000664) were injected with Sugen (SU5416) 20 mg/kg or left untreated (controls) and placed in 10% oxygen or left at room air (controls). At days 8–21, the experimental group was treated with T-5224 30 mg/kg or vehicle control (PVP) for 10 days daily by oral gavage. At 21 days, mice underwent right heart catheterization and were euthanized, and then lungs were fixed in formalin, and single-cell gene expression was analyzed by FLEX scRNA-Seq (10x Genomics).

### Bulk ATAC-Seq processing and data analysis.

ATAC-Seq libraries were prepared as described by Omni-ATAC (56). First, 50,000 fresh primary ECs were pelleted and washed with 1× PBS and 50 μL of chilled lysis buffer (10 mM Tris-HCl, 10 mM NaCl, 3 mM MgCl_2_, 0.1% NP40, 0.1% Tween-20, and 0.01% Digitonin). Next, nuclei were pelleted at 500*g* for 10 minutes at 4^o^C and resuspended in 50 μL of transposition mix containing 1× TD buffer and 100 nM transposase (Illumina Tagment DNA enzyme and buffer small kit, 20034197) to tagment open chromatin. The reaction was incubated at 37°C for 30 minutes at 1,000 rpm on an orbital shaker. Tagmented DNA was purified using DNA Clean and Concentrator-5 kit (Zymo, D4014) and amplified 5–11 cycles using NEBNext 2× MasterMix (New England BioLabs, M0541S). The cycle number for further amplification was determined using a test qPCR. ATAC-Seq libraries were then purified using the Zymo DNA Clean and Concentrator-5 kit and quantified using KAPA library quantification kit (Roche, KK4854). The libraries were paired-end sequenced on the NovaSeq 6000 platform at a depth of 50 million reads per sample. Quality control on the raw reads was carried out using fastqc, and the sequences were aligned to human hg38 reference using Bowtie2 (57). Peaks were called using MACS2 (58), and differential accessible regions were quantified using DiffBind (59) with default parameters.

### Single-cell multiome sequencing nuclei isolation and data analysis.

Lung tissues were dissociated as previously described (17), and transposition of nuclei and library preparation were carried out as per the Chromium Next GEM Single Cell Multiome ATAC + Gene Expression kit protocol (10x Genomics, 1000283). The libraries were sequenced using the Illumina NovaSeq 6000 platform. Raw sequenced data were converted to fastq format using CellRanger’s mkfastq function, and the reads were aligned to GRCh38 (hg38) reference.

Cells with ATAC reads of 100,000 or more or 1,000 or less, RNA reads of 50,000 or greater, ratio of mononucleosomal to nucleosome-free fragments of 2 or higher, TSS enrichment score of 1 or less, or percentage of mitochondrial transcripts of 25% or higher were removed. Peak calling was performed on all filtered cells using MACS2, as implemented in Signac’s CallPeaks function. Gene expression data were log-normalized and scaled using Seurat’s NormalizeData and ScaleData functions. PCA was performed with Seurat’s RunPCA function using variable features determined with Seurat’s FindVariableFeatures function. Term frequency-inverse document frequency (TF-IDF) normalization and singular value decomposition (SVD) were performed on the DNA accessibility data using Signac’s RunTFIDF and RunSVD functions. Harmony (15) was run on the PCA and SVD embeddings separately to correct batch effects between samples. A joint neighbor graph was built with the Harmony embeddings of the gene expression and DNA accessibility count matrices using Signac’s FindMultiModalNeighbors function. Visualization and clustering were performed using Signac’s RunUMAP and FindClusters functions. Cell-type identities were determined using cluster-specific gene markers generated with the FindMarkers function in Seurat. Clusters with a high percentage of mitochondrial transcripts and low RNA and ATAC counts were considered low-quality. Peak calling was performed again on each individual cluster using MACS2, as implemented in Signac’s CallPeaks function. The resulting peak matrix was used subsequently for GRN analysis.

For reclustering of endothelial and mesenchymal clusters, we subsetted the following clusters: 1-Venous Endothelial, 3-Adventitial Fibroblast, 8-Arterial/Capillary Endothelial, 9-Myofibroblast/Interstitial Fibroblast, 11-Lymphatic Endothelial, 15-Smooth Muscle/Pericyte, 21-Endothelial_SC525, and 23-Endothelial_SC527, and performed normalization, dimensionality reduction, batch effect removal, weighted nearest-neighbor graph construction, and clustering and cell-type annotation as described above. Clusters expressing gene markers for 2 or more cell types were excluded from downstream analysis. Motif activity scores were calculated using chromVAR (19), as implemented in Signac’s RunChromVAR function. To identify motifs enriched in SSc vascular ECs, the Wilcoxon rank-sum test was performed on motif activity scores in SSc and healthy control vascular ECs using Seurat’s FindMarkers function.

### Construction of GRNs using SCENIC^+^.

RNA and ATAC count matrix for ECs were subsetted for GRN analysis. Latent Dirichlet allocation models were run on the subsetted ATAC count matrix using pycisTopic (60) to optimize region-topic and topic-cell probability distributions. A model of 60 topics was selected. Binarized region-topic probabilities and differentially accessible regions for each previously identified endothelial and mesenchymal subpopulation were used to infer candidate enhancer regions. Motif enrichment analysis was performed on the candidate enhancer regions using pycistarget with cistarget’s precomputed motif score database (retrieved from https://resources.aertslab.org/cistarget/). Enhancer-driven GRNs (eGRNs) were built using SCENIC^+^ (20). Briefly, target regions of each TF derived from the motif enrichment analysis were extracted to create cistromes. Region-gene and TF-gene relationships were assessed using Gradient Boost Machines. eRegulons (TF-region-gene triplets) were generated using target regions of a TF and genes associated with these regions. The target genes were further filtered based on TF-gene relationships using GSEA. Only eRegulons showing positive region-to-gene correlation were retained. Extended eRegulons were kept only if there was not an available direct eRegulon. Enrichment scores of target regions and target genes were calculated for each eRegulon as AUC at 5% of the ranking. Target region enrichment scores for each cell in the vascular EC cluster were used as input for clustering with Seurat. Briefly, enrichment scores were log-normalized and scaled as previously described. Dimensionality reduction with PCA was performed, followed by correction of sample-wise batch effects with Harmony and visualization with UMAP. Correlations between TF expression and eRegulon target region enrichment scores were assessed to identify high-quality eRegulons. To minimize noise, pseudo-bulks comprising 5 cells sampled from each cell type in SSc or control lungs were generated and used to recalculate eRegulon enrichment scores. eRegulons with correlation coefficients greater than 0.5 or less than –0.5 were retained. Differential expression analysis of target genes was performed using a Wilcoxon rank-sum test as implemented in Seurat’s FindMarkers function. Differential accessibility analysis of target regions was also performed using Seurat’s FindMarkers function (method = LR, latent variable = nCount_peaks_cluster).

### In silico perturbation of genes using CellOracle.

Single-cell ATAC-Seq count matrix for ECs was used as an input to build a base GRN. Using Cicero’s (61) make_cicero_cds function, the *cis*-regulatory or co-accessible pairs of DNA interactions from the single-cell ATAC-Seq peaks were identified. The human hg38 genome was used as a reference gene. The DNA sequences in the peaks were then scanned for TF motif binding using gimme.vertebrate.v5.0 database (62). *Cis*-regulatory peaks with a motif score less than 10 were filtered out, and a base GRN was constructed. scRNA-Seq data from ECs was filtered to remove nonvariable genes, and the top 3,000 variable genes were used for further analysis. ECs were grouped based on health status (healthy or SSc-ILD-PH). Health status–specific GRNs were constructed using CellOracle’s get_links function. To infer these GRNs, a Bayesian ridge regression model was used to provide network edge strength as a distribution. The edges were then filtered on *P* value less than 0.001 to remove weak and uncertain network edges, and the top 2,000 edges for each GRN were picked. The calculated GRN was then used for constructing a predictive model that can be used for perturbation simulations. For KO simulations, the gene expression was set to 0 and for overexpression simulations, the gene expression was set to 1.5 times the maximum gene expression.

### Pseudo-bulk single-cell ATAC-Seq file generation.

Fragment files containing the position of the Tn5 integration site along with the cell barcodes for ECs were extracted from the single-cell multiome experiment using Signac. Single-cell ATAC-Seq BAM files for ECs were subsetted from the entire single-cell multiome dataset using Sinto’s filterbarcodes -b function on the fragment file (https://timoast.github.io/sinto). Peak files were generated on the bam file using MACS2.

### ChromBPNet model training and contribution score generation.

To generate a Tn5 bias model using each sample’s IDR reproducible ATAC-Seq peak file, the chrombpnet bias pipeline function was used with default parameters. Bias model performance was assessed using pearsonr in peaks greater than –0.3, pearsonr in non-peaks greater than 0, and a higher median normalized Jensen Shannon Divergence score. The motifs generated from the Tn5 bias model resembled the Tn5 bias enzyme motif. Using the trained Tn5 bias model, a bias factorized ChromBPNet model was trained using the chrombpnet pipeline function with default parameters. The performance of the ChromBPNet model was assessed using pearsonr in peaks greater than 0.5, a lower mean squared error, and a higher median normalized Jensen Shannon Divergence score. Motifs generated from the ChromBPNet model did not resemble the Tn5 bias enzyme motif. This indicates that the ChromBPNet model was corrected for Tn5 bias. Contribution score bigwigs were generated using chrombpnet contribs_bw function on the counts head generated from the trained ChromBPNet model.

### Clustering of contribution scores and heatmap generation.

The outputs of the contribution count scores were converted to bedGraph files using UCSC Genome Browser’s bigWigToBedGraph function, and the bedGraph files were intersected with the original ATAC peak files generated using BEDTools (63). To identify TF binding sites from ATAC-Seq data, peaks were called on the intersected bedGraph file using MACS3 callpeak function with default parameters. The peaks were then intersected with JASPAR motif coordinates, and the contribution score for each nucleotide was extracted. K-means clustering of the peaks was carried out using the deepTools (64) computematrix and plotheatmap functions with default parameters.

### Statistics.

*P* values for differentially expressed genes were adjusted (*P*_adj_) using a Bonferroni correction by Seurat and considered significant for an adjusted *P* value less than 0.05. Hypergeometric comparisons between gene lists and Monte Carlo simulations were considered significant if *P* was less than 0.05.

### Study approval.

The University of Pittsburgh IRB approved procedures involving human samples. All animal studies were approved by the IACUC of the University of Pittsburgh and were conducted in accordance with the NIH *Guide for the Care and Use of Laboratory Animals* (National Academies Press, 2011) and applicable institutional guidelines.

### Data availability.

The human multiome data are deposited in the NCBI’s Gene Expression Omnibus (GEO GSE302151). The murine scRNA-Seq data are deposited in GEO (GSE314055). Values for all data points in graphs are reported in the Supporting Data Values file.

## Author contributions

RB and YZ contributed equally to this work. RB, YZ, SYC, and RL conceived and designed the study. RB, YZ, MT, and YT performed experiments and generated data. PHG, YZ, RB, JF, JD, AMG, TT, MH, and HS contributed to computational and bioinformatic analyses. SYC and EV provided human samples and clinical expertise. YT, MT, FR, EP, AH, OD, GK, and SYC contributed to mouse studies and data interpretation. RB, YZ, and RL analyzed the data and wrote the manuscript. All authors contributed to editing the manuscript and approved the final version.

## Conflict of interest

RL reports grants from Bristol-Myers Squibb, Formation, Moderna, Regeneron, and Pfizer. RL served or serves as a consultant with AbbVie, Mediar Therapeutics, Bristol-Myers Squibb, Formation, Thirona Bio, Sanofi, Boehringer-Ingelheim, Merck, Genentech/Roche, EMD Serono, Morphic, Third Rock Ventures, Bain Capital, and Zag Bio. RL sits on an independent data safety monitoring committees for Advarra/GSK and Genentech. RL holds stock in and is president of Modumac Therapeutics Inc. SYC has served as a consultant for Merck, Janssen, and United Therapeutics. SYC is a director, officer, and shareholder in Synhale Therapeutics and Amlysion Therapeutics. SYC holds research grants from United Therapeutics, Bayer, and WoodNext Foundation. SYC has filed patent applications regarding the targeting of metabolism and inflammation in pulmonary hypertension (US 10,925,869 B2; US20250295657 A1; PCT/US2015/029286; PCT/US2018/062013; PCT/US2020/059969; PCT/US2021/058511).

## Funding support

This work is the result of NIH funding, in whole or in part, and is subject to the NIH Public Access Policy. Through acceptance of this federal funding, the NIH has been given a right to make the work publicly available in PubMed Central.

- NIH (National Institute of Arthritis and Musculoskeletal and Skin Diseases grant 1P50AR080612).
- NIH grants R01 HL124021 and HL151228 as well as a WoodNext Foundation grant (to SYC).

## Supplementary Material

Supplemental data

Supplemental tables 1-17

Supporting data values

## Version 1 04/22/2026

Electronic publication
