# Supplementary material for: FOSL2 regulates endothelial cell state and chromatin accessibility in systemic sclerosis pulmonary vascular remodeling
Source: JCI Insight. 2026 Apr 22;11(8):e189107. doi: 10.1172/jci.insight.189107 (PMC13135392; doi:10.1172/jci.insight.189107)
Supplement: Supplemental data [file jciinsight-11-189107-s234.pdf]

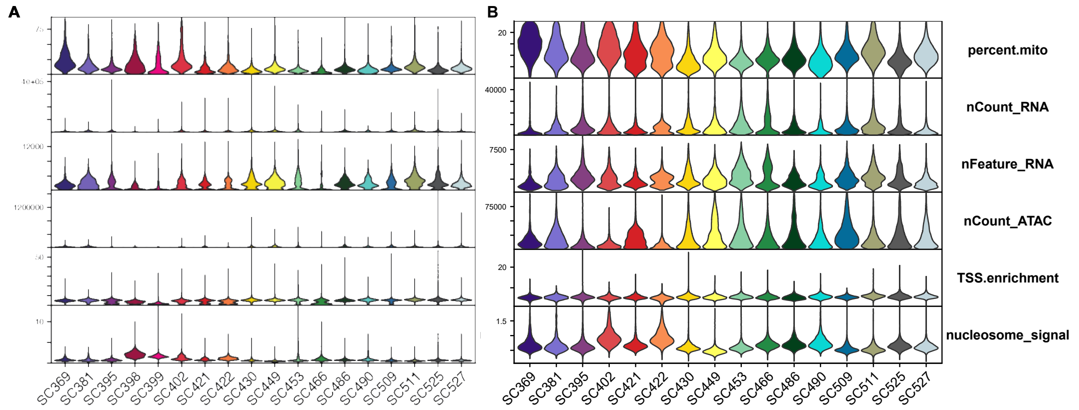

Supplemental Figure 1. Quality control for the single-cell multiome sequencing dataset of SSc and normal lungs.

Percentage of mitochondrial transcripts ("percent.mito"), number of RNA unique molecular identifier (UMI) counts ("nCount_RNA"), number of genes detected (nFeature_RNA), number of ATAC read counts (nCount_ATAC"), transcription start site (TSS) enrichment scores ("TSS.enrichment"), and ratio of mononucleosomal to nucleosome-free fragments ("nucleosome_signal") per cell before (A) and after (B) filtering are shown for each sample.

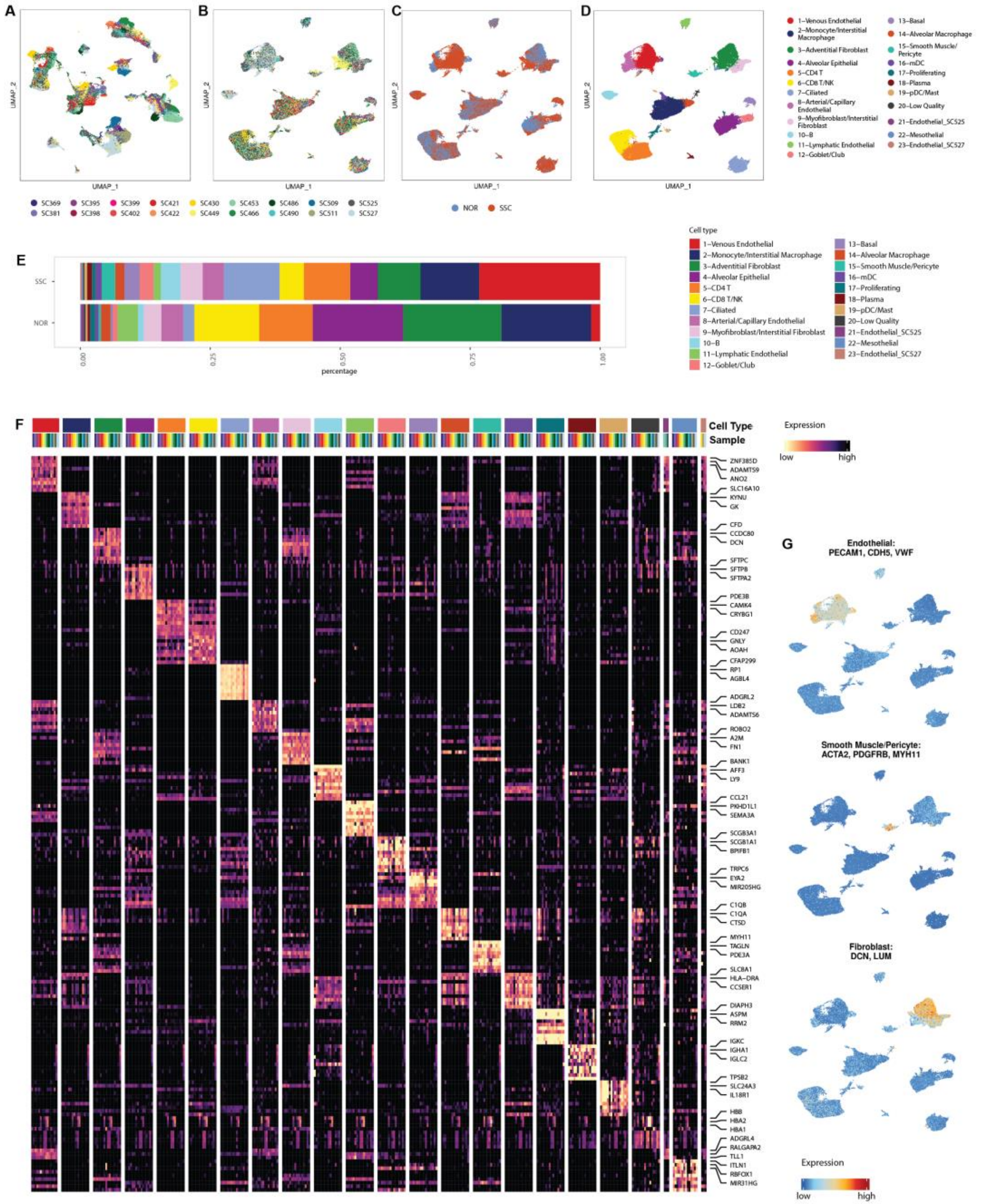

Supplemental Figure 2. Identification of cell types in SSc and normal lung samples using single-cell multiome sequencing.
Panels A-B. UMAP visualization of cells grouped by sample ID with (B) or without (A) integration using Harmony.
Panels C-D. UMAP visualization of cells grouped by (C) health status or (D) cell types.
Panel E. Bar plots showing the percentage of each cell population from normal and SSc lungs.
Panel F. Heatmap showing scaled expression of top 10 gene markers for each cell population ranked by fold change of average expression. Columns are grouped by cell type and each column represents the average expression level for one sample.
G. Expression patterns of gene markers used for identification of the endothelial, smooth muscle/pericyte, and fibroblast clusters.

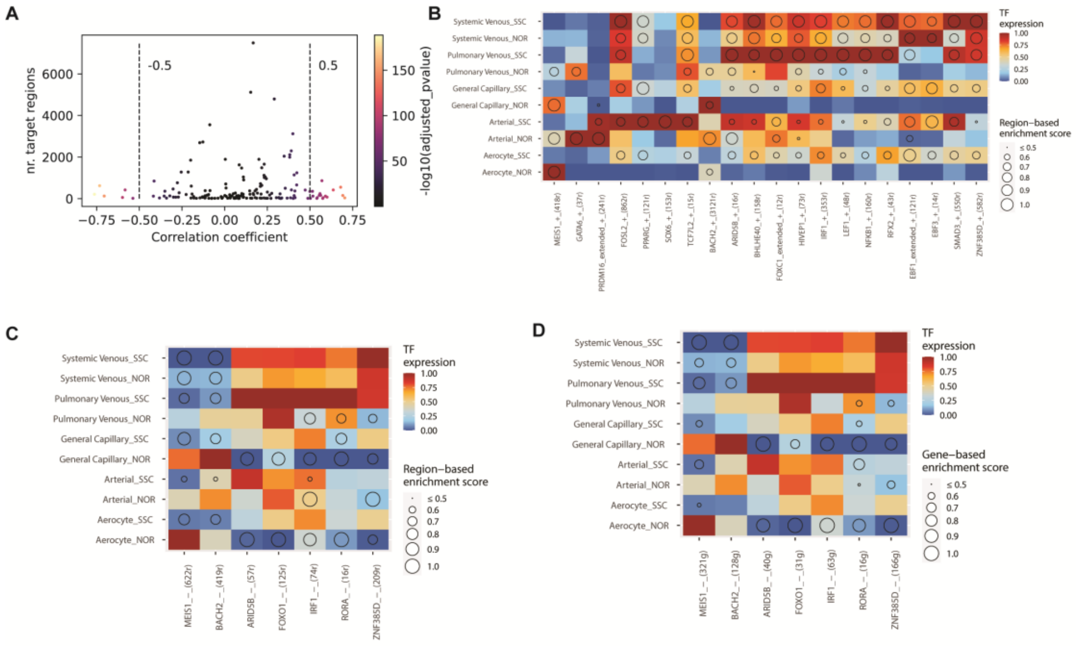

Supplemental Figure 3. SCENIC+ analysis of endothelial and mesenchymal populations.

Panel A: Number of target regions and correlation between expression and target region enrichment scores of each TF. eRegulons with correlation coefficient > 0.5 were identified as high quality and retained for downstream analysis.

Panel B: TF expression (color) and region- based enrichment scores (dot size) for high-quality activator eRegulons.

Panels C-D: TF expression (color) and region-based (C) or gene-based (D) enrichment scores (dot size) for high-quality repressor eRegulons.

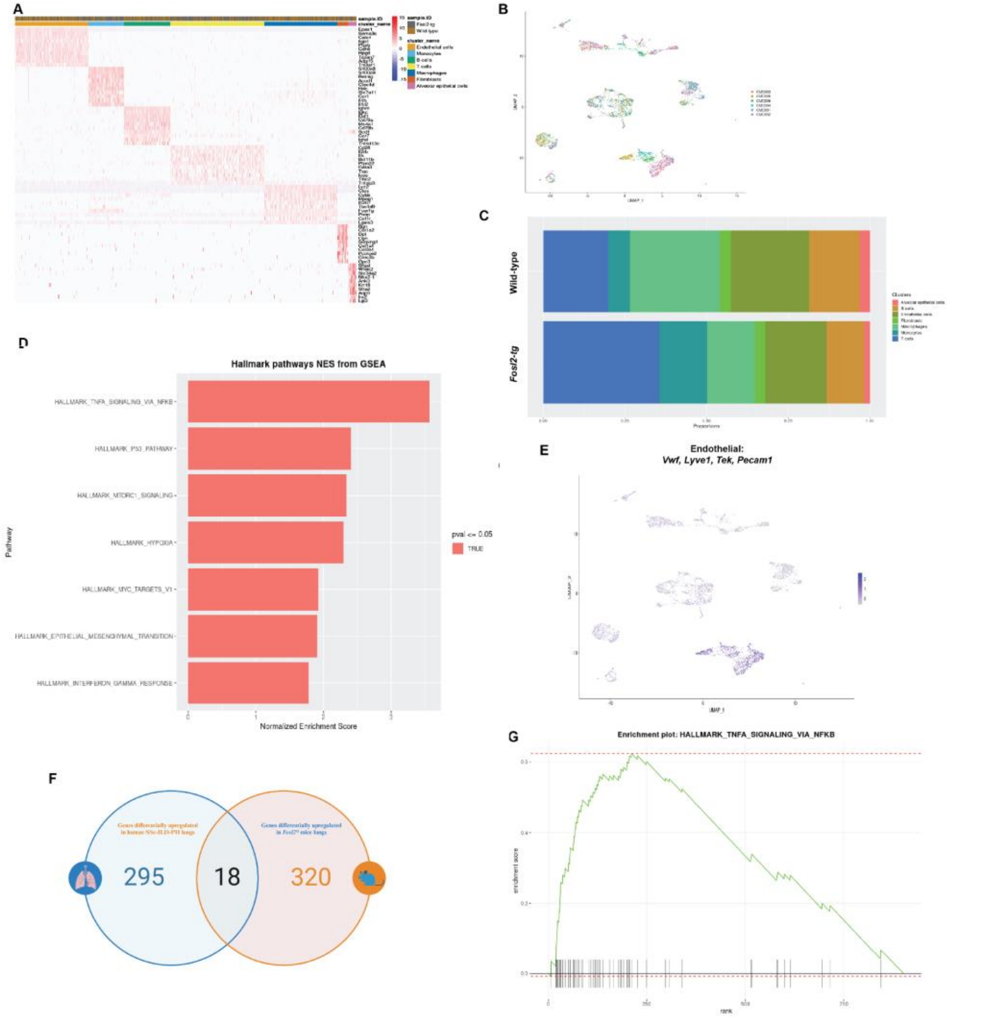

Supplementary Figure 4. Single cell RNA seq analysis on Fosl2-tg and wild-type lungs. Panel A: Markers used to identify cell types from the lungs of Fosl2tg and WT mice. Panel B: UMAP dimensional reduction visualization of cells isolated from each mouse lung sample. CMO301-303 (wild-type), CM304-CM306 (Fosl2-tg). Panel C: Proportion of cells in each cluster identified from wild type and Fosl2tg mice. Panel D: Top Hallmark pathways gene sets (padj<0.05) and their Normalized Enrichment Score (NES) identified by GSEA. Pane E: Feature Plot of genes used to identify vascular endothelial cells from Fosl2tg and wild-type mice

Panel F: Venn diagram of differentially upregulated genes (average Log2Fold Change >0.32 and padj<0.05) shared between endothelial cells from human SSc-ILD-PH patients and Fosl2tg mice. The p value associated with finding 18 genes in common between the upregulated genes in mouse and human datasets is 1.417* 10^ -6 (Exact hypergeometric test). Panel G: Enrichment plot generated of TNFA signaling via NFKB.

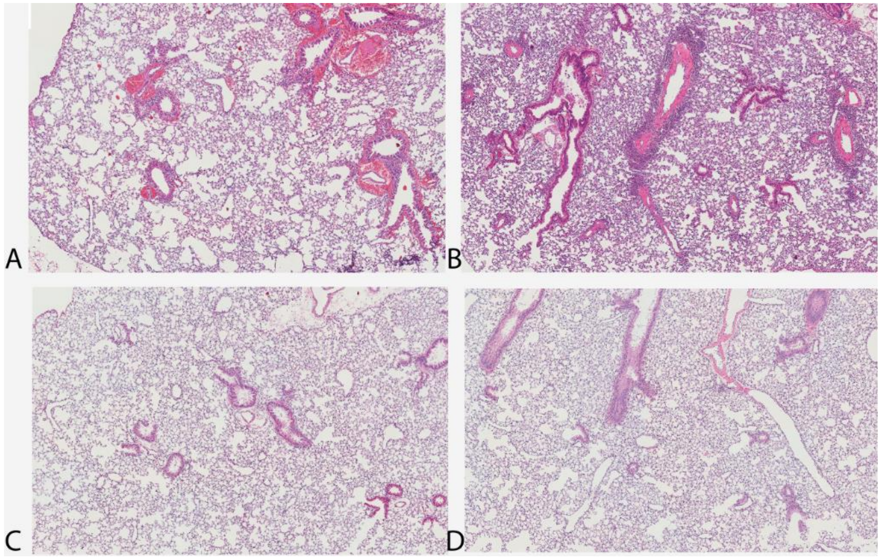

Supplementary Figure 5. Histopathology of early disease WT (panel A), Fosl2^{tg} (panel B), Rag2ko (Panel C) and Fosl2^{tg} rag2ko (panel D).

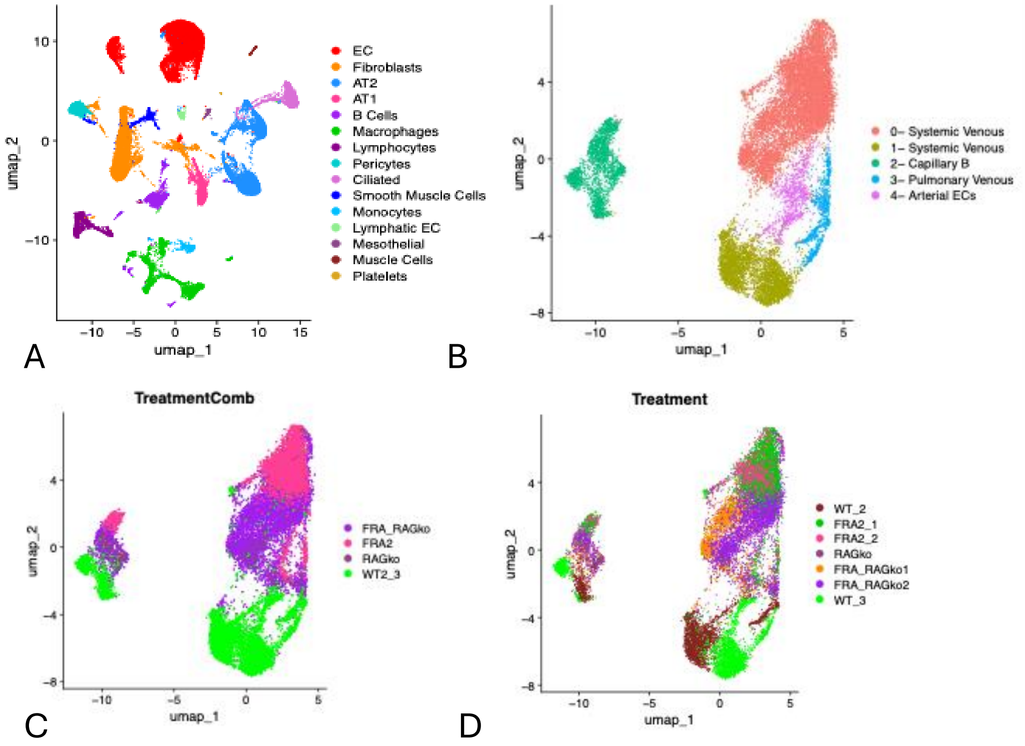

Supplementary Figure 6. UMAP showing all cell clusters (panel A); EC subclusters by EC subpopulation (panel B); EC subclusters by mouse genotype (panel C); and EC subclusters by sample (panel D).

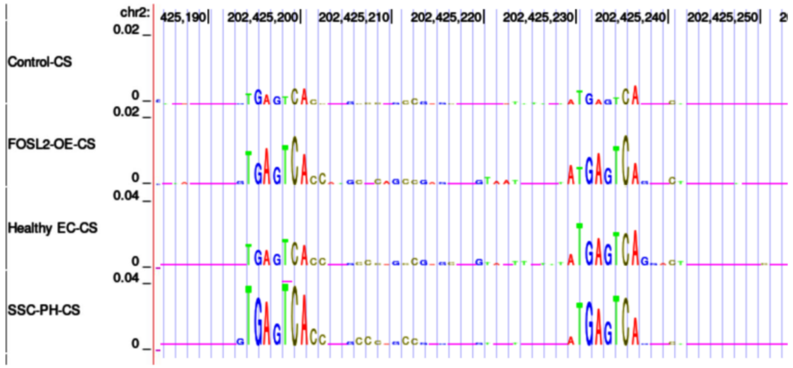

Supplemental Figure 7. Inferred binding to AP-1 enhancer elements is increased the BMPR2 first intron in SSc-ILD-PH and in FOSL2 overexpressing endothelial cells.
